# Transcriptional stimulation of rate-limiting components of the autophagic pathway improves plant fitness

**DOI:** 10.1093/jxb/ery010

**Published:** 2018-01-20

**Authors:** Elena A Minina, Panagiotis N Moschou, Ramesh R Vetukuri, Victoria Sanchez-Vera, Catarina Cardoso, Qinsong Liu, Pernilla H Elander, Kerstin Dalman, Mirela Beganovic, Jenny Lindberg Yilmaz, Sofia Marmon, Lana Shabala, Maria F Suarez, Karin Ljung, Ondřej Novák, Sergey Shabala, Sten Stymne, Daniel Hofius, Peter V Bozhkov

**Affiliations:** 1 Department of Molecular Sciences, Uppsala BioCenter, Swedish University of Agricultural Sciences and Linnean Center for Plant Biology, Uppsala, Sweden; 2 Department of Plant Biology, Uppsala BioCenter, Swedish University of Agricultural Sciences and Linnean Center for Plant Biology, Uppsala, Sweden; 3 Department of Plant Protection Biology, Swedish University of Agricultural Sciences, Alnarp, Sweden; 4 Department of Plant Breeding, Swedish University of Agricultural Sciences, Alnarp, Sweden; 5 ScanBiRes AB, Alnarp, Sweden; 6 School of Land and Food, University of Tasmania, Private Bag, Hobart, TAS, Australia; 7 Departamento de Biologia Molecular y Bioquimica, Facultad de Ciencias, Universidad de Malaga, Campus de Teatinos, Malaga, Spain; 8 Umeå Plant Science Centre, Department of Forest Genetics and Plant Physiology, Swedish University of Agricultural Sciences, Umea, Sweden; 9 Laboratory of Growth Regulators, Centre of the Region Haná for Biotechnological and Agricultural Research, Institute of Experimental Botany Academy of Sciences of the Czech Republic (AS CR), Olomouc, Czech Republic; 10 Faculty of Science, Palacký University, Olomouc, Czech Republic; 11 University of Essex, UK

**Keywords:** Aging, *ATG* genes, autophagy, autophagy-related ubiquitin-like conjugation systems, biomass, oil content, rate-limiting components of autophagic flux, seed yield, stress resistance, transcriptional regulation

## Abstract

Autophagy is a major catabolic process whereby autophagosomes deliver cytoplasmic content to the lytic compartment for recycling. Autophagosome formation requires two ubiquitin-like systems conjugating Atg12 with Atg5, and Atg8 with lipid phosphatidylethanolamine (PE), respectively. Genetic suppression of these systems causes autophagy-deficient phenotypes with reduced fitness and longevity. We show that Atg5 and the E1-like enzyme, Atg7, are rate-limiting components of Atg8–PE conjugation in Arabidopsis. Overexpression of *ATG5* or *ATG7* stimulates Atg8 lipidation, autophagosome formation, and autophagic flux. It also induces transcriptional changes opposite to those observed in *atg5* and *atg7* mutants, favoring stress resistance and growth. As a result, *ATG5*- or *ATG7*-overexpressing plants exhibit increased resistance to necrotrophic pathogens and oxidative stress, delayed aging and enhanced growth, seed set, and seed oil content. This work provides an experimental paradigm and mechanistic insight into genetic stimulation of autophagy *in planta* and shows its efficiency for improving plant productivity.

## Teaser

Autophagy-related proteins Atg5 and Atg7 are rate-limiting components of autophagic flux in Arabidopsis. Overexpression of *ATG5* or *ATG7* genes stimulates Atg8 lipidation, autophagosome formation, and autophagic flux, leading to improved plant fitness.

## Introduction

Homeostasis of all biological systems relies on the continuous renewal of individual subunits. The turnover of cellular components ensures the replacement of old or damaged macromolecules and organelles by new ones. Autophagy is a major catabolic process in eukaryotic cells, able to degrade not only proteins and protein complexes but also entire organelles. Upon induction of autophagy, autophagic cargo is sequestered into double membrane vesicles, autophagosomes, and digested following fusion of autophagosomes with lysosomes or lytic vacuoles (Mizushima and Komatsu, 2011; Klionsky *et al.*, 2016). The dynamic process of autophagosome formation, delivery of autophagic cargo to the lysosome or vacuole, and degradation defines autophagic flux, which can be measured experimentally by a number of dedicated assays.

Autophagy plays a paramount role in eukaryotic life as a key process maintaining proteostasis, conferring stress tolerance, and suppressing aging (Levine *et al.*, 2011; Mizushima and Komatsu, 2011; Rubinsztein *et al.*, 2011). Under favorable conditions, a low level of autophagic flux serves housekeeping functions by clearing obsolete cytoplasmic content. During periods of stress or starvation, autophagic flux is enhanced to promote cell survival by recycling damaged proteins and organelles and thereby reallocate energy and building blocks for the biosynthetic processes (Rabinowitz and White, 2010; Mathew and White, 2011).

Autophagy-related (*ATG*) genes were first discovered in budding yeast and later shown to be conserved in almost all eukaryotes (Yang and Klionsky, 2010). To date, >35 *ATG* genes are functionally characterized in yeast, and most of them have close homologs in plants (Liu and Bassham, 2012; Shibutani and Yoshimori, 2014). Understanding the role of autophagy in plant biology was largely facilitated by the use of *ATG*-knockout (*atg*) mutants of *Arabidopsis thaliana*. At the cellular level, autophagy in plants participates in a whole array of vital processes, such as starch degradation (Wang *et al.*, 2013), chloroplast recycling (Xie *et al.*, 2015), elimination of oxidized proteins (Xiong *et al.*, 2007) and peroxisomes (Shibata *et al.*, 2013), salicylic acid signaling (Yoshimoto *et al.*, 2009), cytoprotection against necrosis (Minina *et al.*, 2013*a*), and both initiation and execution of programmed cell death (Minina *et al.*, 2014). At the whole-plant level, these cellular functions of autophagy jointly contribute to an efficient nutrient remobilization (Guiboileau *et al.*, 2012; Guiboileau *et al.*, 2013), stress tolerance (Zhou *et al.*, 2013, 2014), control of senescence (Yoshimoto *et al.*, 2009), disease resistance (Hofius *et al.*, 2009; Lai *et al.*, 2011), and longevity (Minina *et al.*, 2013*b*). Accordingly, decreased autophagic flux in Arabidopsis *atg* mutants correlates with the overall reduction in plant fitness, including reduced growth and fecundity, accelerated senescence, as well as high susceptibility to nutrient starvation, other types of abiotic stresses, and necrotrophic pathogens.

**Fig. 1.**
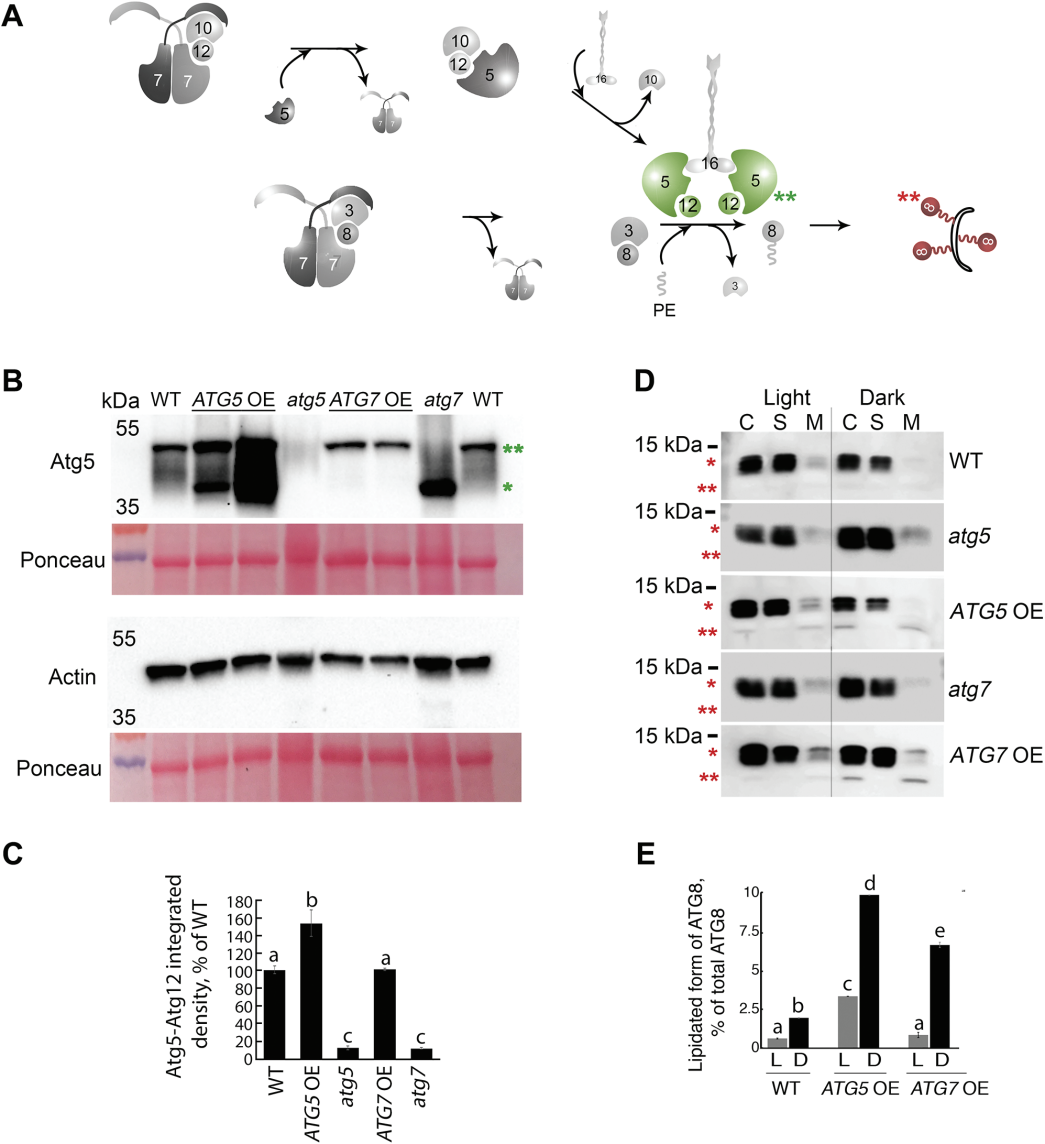
Constitutive overexpression of *ATG5* or *ATG7* in Arabidopsis stimulates lipidation of Atg8. (A) A schematic representation of two autophagy-related ubiquitin-like conjugation systems (Nakatogawa, 2013). Atg7 acts as an E1-like ligase by stimulating conjugation of two substrate pairs: of a ubiquitin-like protein Atg12 with its E2-like ligase, Atg10, and of a ubiquitin-like protein Atg8 with its E2-like ligase, Atg3. Atg12 is further transferred from Atg10 onto Atg5 protein in an E3-ligase-independent manner. The Atg12–Atg5 conjugate forms a complex with Atg16 and gains an E3-like ligase activity. Atg12–Atg5–Atg16 E3-like ligase stimulates conjugation of Atg8 with a phosphatidylethanolamine (PE), followed by anchoring of Atg8–PE on a membrane of a growing phagophore. Double green and double red asterisks indicate Atg12–Atg5 and Atg8–PE conjugates, respectively, also shown on the western blots on (B) and (D). (B) Atg5, but not Atg7, is a limiting component in the Atg12–Atg5 conjugation pathway. Western blot detection of Atg5 in rosette leaves of Col-0 (WT), two individual *ATG5*- or *ATG7*-overexpressing lines (*ATG5* OE or *ATG7* OE, respectively), and *ATG5*- or *ATG7*-knockout (*atg5* or *atg7*, respectively) mutants. *Atg5; **Atg12–Atg5 conjugate. The experiment was repeated four times, using three individual lines of each overexpressor background. Western blot detection of actin and Ponceau staining were used as loading controls. (C) Densitometry of the Atg5–Atg12 conjugate in (B). Integrated density of bands corresponding to the Atg5–Atg12 conjugate were first normalized to the corresponding values for actin and then expressed as a percentage of the obtained value for the WT. Data represent means ±SE; *n*=6. Mean values denoted by the same letter do not differ significantly at *P*<0.001 (Student’s *t*-test). (D) Up-regulation of either *ATG5* or *ATG7* stimulates lipidation of Atg8. Seven-day-old seedlings of Col-0 (WT), *atg5*, *ATG5* OE, *atg7*, and *ATG7* OE genotypes were incubated for 3 d under 150 µE m^–2^ s^–1^ light, 16 h photoperiod (Light) or in the darkness (Dark). Total proteins were fractionated by ultracentrifugation and used for western blot analysis to detect free (*) and lipidated (**) forms of Atg8. C, crude extract; S, supernatant of 100000 *g* fraction; M, pellet of 100000 *g* fraction, containing membranes. (E) Densitometry of the Atg8–PE conjugate in (D). Integrated density of bands corresponding to the Atg8–PE in the crude fraction was expressed as a percentage of the integrated density of the total amount of Atg8 in the corresponding sample. Data represent means ±SE; *n*=6. Mean values denoted by the same letter do not differ significantly at *P*<0.001 (Student’s *t*-test). L, light; D, dark.

The above findings unequivocally illustrate the importance of preventing a decline in the autophagic flux to minimize the impact on growth and stress sensitivity. The question is whether one can achieve an opposite, invigorating effect on plants by increasing autophagic flux. We have previously shown that autophagy can be enhanced in wild-type (WT) Arabidopsis plants by moderately reducing light intensity, conditions that mimic an effect of caloric restriction in animals (Rubinsztein *et al.*, 2011), resulting in suppression of senescence and extension of life span (Minina *et al.*, 2013*b*). Recent studies using budding yeast and animal models have uncovered transcriptional and epigenetic regulation of *ATG* genes as essential mechanisms modulating the autophagic response and maintaining homeostasis necessary for stress tolerance and longevity (Feng *et al.*, 2015; Lapierre *et al.*, 2015). Notably, genes encoding the components of two ubiquitin-like conjugation systems (*ATG3*, *ATG5*, *ATG7*, *ATG8*, *ATG10*, *ATG12*, and *ATG16*) operating to form Atg12–Atg5 and Atg8–phosphatidylethanolamine (PE) conjugates (Fig. 1A; Chen and Klionsky, 2011) are among the most frequently found targets of transcription factors, miRNAs, and chromatin-modifying enzymes (Frankel and Lund, 2012; Füllgrabe *et al.*, 2016). Previous works, including the two most recent phenotypic studies, indicated that ectopic expression of the components of the two autophagy-related conjugation systems have beneficial effects on plant growth and stress tolerance (Slavikova *et al.*, 2008; Xia *et al.*, 2012; Wang *et al.*, 2016, 2017). Although these studies did not provide a mechanistic link between expression of certain *ATG* genes and the regulation of autophagic flux, they suggest that transcriptional activation of the components of the Atg12–Atg5 and Atg8–PE conjugation systems might be the ‘bottleneck’ during autophagy induction.

In the present work, we found that constitutive overexpression of *ATG5* or *ATG7* in Arabidopsis enhances the activity of the two conjugation systems, autophagosome formation and autophagic flux, leading to suppression of aging and strong stimulation of growth and stress resistance. To provide the first insight into the possible molecular mechanisms underlying the phenotypic differences of plants with enhanced and suppressed autophagy, we performed global gene expression analysis, which revealed key transcriptional trends associated with up-regulated or impaired autophagy.

## Materials and methods

### Plant material

We used the previously described T-DNA knockout lines *atg5-1* (Thompson *et al.*, 2005) and *atg7-2* (Hofius *et al.*, 2009) referred to here as *atg5* and *atg7*, respectively. For generation of overexpression lines, cDNA of *ATG5* and coding DNA sequence of *ATG7* were amplified using primers attB1-ATG5UTR-Fw/attB2-ATG5-Rev and FWatg7/RVatg7, respectively (see Supplementary Table S5 at *JXB* online). The PCR products were recombined under the control of the 35S promoter into the pGWB2 vector (Nakagawa *et al.*, 2007) (GenBank accession no. AB289765.1) using the Gateway cloning system (Invitrogen). pGWB2 constructs were used for transformation of *Agrobacterium tumefaciens* strain GV3101. WT Arabidopsis plants of the Col-0 ecotype were transformed using the floral dip method (Clough and Bent, 1998). Transgenic plants were selected on Murashige and Skoog (MS) medium containing 50 µg ml^–1^ kanamycin. Seeds of the homozygous T_3_ generation were used for further experiments.

WT, *ATG5*- or *ATG7*-overexpressing (*ATG5* OE or *ATG7* OE), *atg5*, and *atg7* plants were crossed with a green fluorescent protein (GFP)–Atg8a-expressing line kindly provided by R. Vierstra (Thompson *et al.*, 2005). Crosses were brought to the F_4_ generation to establish homozygous knockout and *ATG*-overexpressing backgrounds, and checked with qPCR to ensure lack of somatic silencing.

The homozygous T_3_ generation of *ATG5* OE and *ATG7* OE lines were reciprocally crossed to obtain *ATG5/ATG7* OE lines. The F_2_ generation of the double overexpressors was used for further experiments. Individual plants were genotyped in each experiment to confirm the presence of both transgenes.

### Plant growth conditions

Seeds were dried at 37 °C for 48 h, treated at –20 °C overnight, surface-sterilized in 10–15% bleach for 10–30 min, and rinsed in sterile deionized water. Sterilized seeds were placed on half-strength MS medium (M0222, Duchefa), supplemented with 1% (w/v) sucrose, 10 mM MES (pH 5.8), and 0.6% (w/v) plant agar (P1001, Duchefa), and vernalized at 4 °C for 48 h. Germination was carried out in growth rooms at 16 h/8 h light/dark cycles, light intensity 110 μE m^–2^ s^–1^, and 22 °C/20 °C day/night temperature. Seedlings with four rosette leaves were transferred into individual 8 cm^3^ pots filled with soil S-Jord (Hasselfors) and grown in controlled-environment cabinets (Percival AR- 41L2, CLF Plant Climatics) at 16 h/8 h light/dark cycles, at 65% relative humidity, 22 °C/20 °C day/night temperature, and light intensity 150 μE m^–2^ s^–1^ at the level of the leaf rosette. Plants were regularly watered with tap water.

### Western blotting

Lipidation of Atg8 was assessed as previously described (Chung *et al.*, 2010). Atg8a was detected using an antibody kindly provided by Y. Ohsumi (Yoshimoto *et al.*, 2004). For NBR1 (neighbor of BRCA1) detection, 100 mg of the sampled plant material was mixed with 100 µl of urea extraction buffer (4 M urea, 100 mM DTT, 1% Triton X-100) and incubated on ice for 10 min. Samples were boiled in two vols of 2× Laemmli sample buffer (Laemmli, 1970) for 10 min and centrifuged in a table centrifuge at 13 000 rpm for 15 min. Equal amounts of supernatants were loaded on a pre-cast 4–20% polyacrylamide gel (Bio-Rad) and blotted onto a polyvinylidene difluoride (PVDF) membrane. Membranes were cut horizontally at the level corresponding to ~55 kDa. The top part of a membrane containing proteins with a mol. wt >55 kDa was used for blotting with anti-NBR1 1:2000 [kindly provided by T. Johansen (Svenning *et al.*, 2011)] and the lower part of the membrane containing proteins <55 kDa was blotted with anti-actin 1:2000 (AS13 2640, Agrisera). The reaction was developed using an ECL Prime kit (RPN2232, Amersham, GE Healthcare) and detected in Chemidoc XRS+ (Bio-Rad). Several exposures were selected for different samples to avoid quantification of saturated signal. Comparisons of absolute integrated density values for each line were made using the same exposure. First, for each exposure, corresponding background values were subtracted from integrated density values of the protein bands. Then the integrated density values for bands corresponding to NBR1 protein were normalized to the respective values of actin for the same sample. Finally, obtained values were expressed as relative values, namely as the percentage of 0 days after the first flower opened (DAF) for each line. For each line, normalized values for NBR1 protein at 0 DAF were assigned as 100% and values for 10 DAF were recalculated as a percentage of it. All images were quantified using ImageJ software.

For GFP–Atg8 cleavage assay, sterilized seeds were sown on 50 µm nylon mesh placed on the top of half-strength MS medium, supplemented with 1% (w/v) sucrose, 10 mM MES (pH 5.8), and 0.6% (w/v) plant agar, and vernalized at 4 °C for 48 h. All genotypes were represented on each plate; four plates were used in each experiment. Plates were incubated vertically in a growth cabinet (Sanyo) at 16 h/8 h light/dark cycles, light intensity 120 μE m^–2^ s^–1^, and 21 °C/20 °C day/night temperature for 7 d. To induce starvation, meshes with seedlings from half of the plates were transferred to half-strength MS medium without sucrose, and plates were wrapped in metal foil and incubated under the same conditions for a further 4 d. Seedling of the same genotype grown under the same conditions were pooled together, ground in liquid nitrogen, boiled in 2 vols of 2× Laemmli sample buffer (Laemmli, 1970) for 10 min, and centrifuged in a table-top centrifuge at 13000 rpm for 15 min. Equal amounts of supernatants were loaded on a pre-cast 4–20% polyacrylamide gel (Bio-Rad), blotted on a PVDF membrane, and stained with anti-GFP (632381, Clontech). The reaction was developed using the ECL Prime kit and detected using Chemidoc XRS+ (Bio-Rad). Several exposures were selected for different samples to avoid quantification of saturated signal. Comparisons of absolute integrated density values for each line were made using the same exposure. For each exposure, corresponding background values were subtracted from integrated density values, then integrated density values for bands corresponding to free GFP were expressed as relative values, namely as a percentage of total GFP present in the same sample. For each line, a sum of values for free GFP and GFP–ATG8a was assigned as 100% and values for free GFP were recalculated as a percentage of it. All images were quantified using ImageJ software.

For Atg5 detection, plant material was ground in liquid nitrogen, mixed with 2 vols of 2× Laemmli buffer, and boiled for 10 min. Debris was pelleted for 5 min at 17000 *g*. Proteins were separated on a 10% polyacrylamide gel, transferred onto PVDF membranes, and blotted with anti-Atg5 1:1000 [kindly provided by R. Vierstra (Thompson *et al.*, 2005)] or anti-actin 1:2000 (AS13 2640, Agrisera). Detection of Atg5 and actin was performed on two separate membranes due to the similarity in molecular weight of the proteins of interest. The reaction was developed using the ECL Prime kit and detected using LAS-3000 (Fujifilm). All membranes were additionally stained in Ponceau to visualize the total protein concentration in the samples.

### Plant growth analysis

Plant growth analysis was performed according to the previously described procedure (Minina *et al.*, 2013*b*). Values outside the range of ± double the SD were considered outliers and were excluded from the statistical analysis. Each growth trial included WT, *ATG5* OE or *ATG7* OE lines, *atg5* or *atg7* mutants, and sometimes the corresponding complementation lines, with 20–40 plants per genotype, and was repeated twice, every time in a different growth cabinet. Plants of different genotypes were randomly distributed in the growth cabinets. For estimating seed set, seeds were harvested from 6–11 individual plants per genotype using an ARACON device (Arasystem), sieved, and weighed on ultra-balances. For life span determination, plants were considered dead at the stage after rosettes underwent senescence and degradation, and new inflorescences, leaves, flowers, or flower buds were no longer emerging, indicating cessation of cell division in the shoot apical meristem.

### Microscopy

Six-day-old seedlings grown on half-strength MS medium, supplemented with 1% (w/v) sucrose, 10 mM MES (pH 5.8), and 0.6% (w/v) plant agar were transferred into liquid half-strength MS medium containing 0.5 µM concanamycin A (ConA; C9705, Sigma), briefly vacuum infiltrated, and left under light for 16 h before imaging.

Root epidermal cells of the elongation zone were imaged using an LSM 780 confocal microscope (Carl Zeiss), 488 nm argon laser, and ×63 objective (NA1.2, water immersion). One to three cells per root and 4–7 roots per genotype were imaged for the analysis. The images were processed using ImageJ 1.51g (Fiji) to measure the number and area of GFP puncta in the cell vacuoles. The mean number of GFP–Atg8 puncta per cell of each genotype was compared with that of Col-0 by performing a Dunnett’s test and assuming a Poisson distribution of the data (‘Multcomp’ R package; Hothorn *et al.*, 2008). The mean areas of the puncta per genotype were compared using the Wilcoxon rank sum test with continuity correction in R.

### Pathogen infections

For necrotrophic fungal infection, *Alternaria brassicicola* strain MUCL20297 was cultured on potato dextrose agar plates for 2 weeks at 22 °C. Spores were harvested in water and filtered through Miracloth (EM475855-1R, VWR) to remove hyphae. The spore suspension was adjusted to the final concentration of 5 × 10^5^ spores ml^–1^ supplemented with 0.05% Tween-20. *Alternaria brassicicola* inoculation of 3-week-old plants was performed by adding 10 µl drops of spore suspension onto the upper leaf surface as described previously (Thomma *et al.*, 1998). Plants were maintained under saturating humidity for 1 d prior to pathogen inoculation and 2 d post-inoculation. Leaf samples for fungal quantification were collected 7 d post-inoculation, snap-frozen in liquid nitrogen, and stored at –70 °C prior to DNA extraction. Total DNA was extracted from frozen leaf samples using the GeneJET Plant Genomic DNA Purification Kit (K0791, Thermo Fisher Scientific) following the manufacturer’s protocol. Fungal DNA quantification of three independent biological replicates was carried out by quantitative real-time (qRT)-PCR using the iQ5 qPCR System (Bio-Rad) to detect fungal *cutinase* (GI 416217) and Arabidopsis *UBQ5* (AT3G62250) and *PR2* (AT3G57260) genes with corresponding primer pairs listed in Supplementary Table S5.

### Oxidative stress and chlorophyll measurement

Seeds were sown on half-strength MS medium as described above, with or without addition of 0.1 μM methyl viologen (MV; 856177, Sigma) and grown in vertically positioned plates. For each genotype, several seedlings were pooled into three groups representing biological replicates. Pooled seedlings were weighed and incubated in 80% acetone overnight at 4 °C in the darkness. A 150 μl aliquot of each chlorophyll extract was used to measure absorbance at 647 nm and 665 nm. Chlorophyll content was estimated as described previously (Inskeep and Bloom, 1985) and normalized to the fresh weight of the corresponding sample.

### Rosette leaf number and cell size measurements

Seeds were dried at 37 °C for 48 h, treated at –20 °C overnight, surface-sterilized in 10–15% bleach for 10–30 min, and rinsed in sterile deionized water. Sterilized seeds were sown directly into soil and grown in controlled environment rooms at 16 h/8 h light/dark cycles, 22 °C/20 °C day/night temperature, and light intensity 150 μE m^–2^ s^–1^ at the level of the leaf rosette. Three individual lines overexpressing *ATG5* or *ATG7* were used for the analysis to exclude possible insert position effects. At least four biological replicates were used for each genotype. All genotypes were represented in each tray and placed at random positions. Plants were regularly watered with tap water and imaged every 2–3 d.

Most bottom rosette leaves were sampled at 20 DAF to ensure full expansion of the leaf blade. Leaf samples were treated as described previously (Wuyts *et al.*, 2010). Leaf blade areas closest to petioles were imaged from the abaxial side using Axioplan A1 (Carl Zeiss) and ZEN lite software. For area measurement, individual epidermal cells were selected using the freehand selection tool of ImageJ software and a touchscreen. Eight to 10 cells per image, at least three images per plant, and three plants per genotype were analyzed.

### Transcriptome profiling

Plants were grown at 16 h/8 h light/dark cycles, 120 μE m^–2^ s^–1^ light intensity, and 22 °C in individual pots. All genotypes were represented in each tray and were placed at random positions. Complete rosettes were sampled at the budding stage and 10 DAF. Four biological replicates were pooled together for each genotype. The material was stored at –80 °C prior to RNA extraction.

RNA was extracted from the material ground in liquid nitrogen using a Spectrum Plant total RNA kit (STRN250, Sigma) and treated with Turbo DNase (AM2238, ThermoFisher). Quality and concentration of RNA were analyzed with NanoDrop and BioAnalyzer. Samples with an RNA integrity number (RIN) >6 were used for further analysis. The expression level of *ATG5* and *ATG7* was verified for all samples by qRT-PCR analysis (data not shown).

The gene expression assay was done on an Agilent 8 × 60 K ArrayXS, and primary normalization and quality control of data were performed at OakLabs, Germany (for more information, see Supplementary file S1). Common trends in changes of transcriptional profiles for both *ATG5* OE and *ATG7* OE lines were compared with WT and both knockout genotypes. Because *ATG5* OE and *ATG7* OE or knockout genotypes were pooled together for the analysis, a fold change >1.5 was considered as significant and a *P*-value <0.1 acceptable.

Venn diagrams were built in Venny 2.1.0 to see intersects between common differentially expressed genes (DEGs). The obtained lists of targets were used for gene ontology using Virtual Plant 1.3 (http://virtualplant.bio.nyu.edu/cgi-bin/vpweb/) and Classification SuperViewer Tool w/ Bootstrap (http://bar.utoronto.ca/ntools/cgi-bin/ntools_classification_superviewer.cgi).

Corresponding 3'-untranslated regions (UTRs) were not included in both constructs used for overexpression (pGWB2 35S::ATG5 and pGWB2 35S:ATG7). Microarray chip used for analysis carried the probe ZA7224578 (TCCTCAAAGGTGAAGTGTAAGGTTCTCTGCAGTTA CAATCCATCTGTGAATTG) complementary primarily to the 3'-UTR of *ATG5* (AT5G17290); thus only endogenous *ATG5* expression levels were detected. ZA7248403 probe (TGATACTGATGATGACGATGTAGCTGTAGATCTTTAAA GACAGATTTAT) annealing mostly on the coding 3' part of *ATG7* (AT5G45900) allowed detection of both endogenous and exogenous *ATG7* transcripts. Microarray data also revealed low levels of *ATG5* and *ATG7* expression in the corresponding knockout lines, consistent with our previous observations obtained by qRT-PCR analysis. Although full-length mRNA of *ATG5* and *ATG7* could not be detected in the corresponding knockout lines, both 5' and 3' parts of the transcripts were detectable, indicating transcription of partial sequences driven from T-DNA insertion promoters.

### Data analysis

Data were analyzed using the JMP 10.0.0 64-bit edition software, unless described differently. If not stated otherwise, Dunnett’s test was used for comparing transgenic lines with the WT. Survival analysis of life span data was performed using the Kaplan–Meier method.

qRT-PCR and analysis of lipid content were performed as described in the Supplementary methods.

## Results

### Overexpression of *ATG5* or *ATG7* stimulates ubiquitin-like conjugation systems and enhances autophagic flux in Arabidopsis

We generated a panel of homozygous transgenic lines of Arabidopsis constitutively overexpressing either *ATG5* or *ATG7* under control of the 35S promoter (*ATG5* OE or *ATG7* OE, respectively), and showing 7- to 10-fold higher transcript levels of the corresponding genes compared with WT (Col-0) plants (Supplementary Fig. S1A). Additionally, we established lines expressing GFP–Atg8a in WT, *ATG5* OE, *ATG7* OE, *ATG5*-knockout (*atg5*), and *ATG7*-knockout (*atg7*) backgrounds (Supplementary Fig. S1B). In agreement with previous studies (Thompson *et al.*, 2005), homologous overexpression of *ATG8a* in Arabidopsis did not reveal any discernible phenotype.

First, we assessed the impact of *ATG5* or *ATG7* overexpression on the activity of the ubiquitin-like conjugations systems (Fig. 1A) by immunodetection of two conjugates generated by the systems: Atg12–Atg5 (Fig. 1B, C) and Atg8–PE (Fig. 1D, E). We found that overexpression of *ATG5* enhanced both Atg12–Atg5 and Atg8–PE conjugation, whereas overexpression of *ATG7* had stimulatory effect only on the lipidation of Atg8 and did not influence the efficacy of Atg12–Atg5 conjugation (Fig. 1B–E). Importantly, overexpression of either *ATG5* or *ATG7* did not affect expression levels of other components of the ubiquitin-like conjugation systems (Supplementary Fig. S2). Collectively, these results indicate that Atg5 and Atg7 are rate-limiting components of the Atg8 lipidation pathway. While Atg5 exerts its effect on Atg8 lipidation via directly controlling the rate of Atg12–Atg5 conjugation (Fig. 1A–C), Atg7 presumably acts via catalyzing formation of the Atg8–Atg3 conjugate (Fig. 1A, D, E).

We attempted to assess a possible impact of a simultaneous overexpression of *ATG5* and *ATG7* genes by crossing single overexpressors. Interestingly, the amount of Atg5–Atg12 conjugate in double overexpressors was similar to the level in the single *ATG5* OE (Supplementary Fig. S3A, B), corroborating the notion that Atg5 but not Atg7 is a rate-limiting component of this reaction. Unfortunately, expression levels of the two genes in double overexpressors were considerably lower than in single overexpressors (Supplementary Fig. S3C), impeding an accurate comparison of the respective phenotypes. Additionally, simultaneous overexpression of both *ATG* genes caused a significant transcriptional down-regulation of some components of the conjugation systems, including *ATG8* isoforms and *ATG12a* (Supplementary Fig. S3C). Although this phenomenon hampers further investigation of the simultaneous expression of *ATG5* and *ATG7*, it provides interesting evidence for a negative feedback loop connecting autophagic flux and transcription of plant *ATG* genes.

**Fig. 2.**
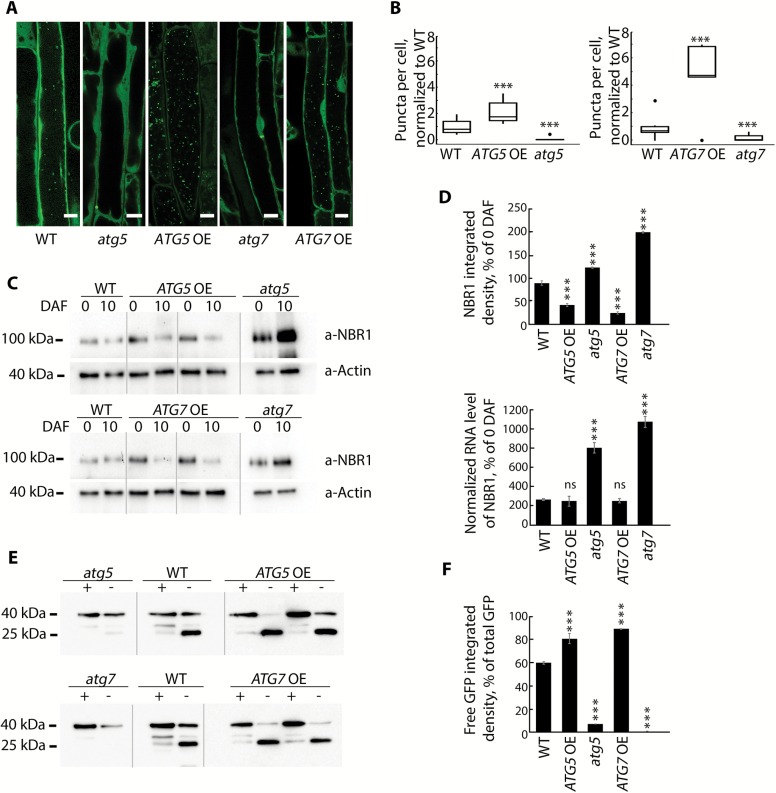
Constitutive overexpression of *ATG5* or *ATG7* stimulates formation of autophagosomes and increases autophagic flux. (A) Representative confocal microscopy images of the epidermal root cells expressing GFP–Atg8a in WT, *ATG*-knockout, and *ATG*-overexpressing backgrounds. Seedlings were grown under 150 µE m^–2^ s^–1^ light, 16 h photoperiod for 6 d and subjected to 0.5 µM ConA treatment for 16 h prior to imaging. Scale bars=10 µm. (B) The box-plot diagram shows the number of the GFP-positive puncta in the epidermal root cells in (A). The average number of puncta for two or three cells belonging to the same root was considered as a single measurement. Four to seven roots were imaged per genotype. The means of each genotype were compared with the WT using Dunnett’s test, assuming a Poisson distribution of the data. ***P<0.001; ∙, outliers. (C) Increase of autophagic flux in *ATG5*- or *ATG7*-overexpressing plants confirmed by a higher rate of NBR1 degradation. Plants of the indicated genotypes were grown under 150 µE m^–2^ s^–1^. Rosette leaves were sampled at the onset of flowering (0 days after flowering, DAF) and 10 d later (10 DAF). Total protein extracts from sampled leaves were used for western blot analysis to detect NBR1 and actin. (D) Densitometry of the NBR1 signal in (C) and expression level of *NBR1* in the same samples. Integrated density values for NBR1 were first normalized to actin and then expressed as a percentage of 0 DAF for the corresponding sample. Data represent means ±SE; *n*=6. The means of each genotype were compared with the WT using Dunnett’s test, ***P<0.001. Detection of the *NBR1* transcript level in *ATG*-overexpressing plants confirmed that the decrease in NBR1 protein was caused by protein degradation and did not originate from transcriptional variation. qRT-PCR was performed on the same leaf material as in (C). Data represent means ±SE for each genotype, normalized to two reference genes (*PP2A* and *HEL*) and to 0 DAF; *n*=6. The means of each genotype were compared with the WT using Dunnett’s test, ***P<0.001. (E) Increased autophagic flux in *ATG5*- or *ATG7*-overexpressing plants additionally confirmed by detection of free GFP accumulation in the seedlings expressing GFP–Atg8a in the indicated backgrounds. Seven-day-old seedlings grown under normal conditions (150 µE m^–2^ s^–1^ light, 16 h photoperiod) were either transferred onto sucrose-depleted MS medium and kept in the darkness (–) or left growing under normal conditions (+). Total protein extracts from the whole seedlings of each genotype were used for western blot analysis with anti-GFP. The GFP–Atg8a fusion has a predicted molecular weight of ~40 kDa; free GFP has a predicted molecular weight of 27 kDa. To avoid quantification of saturated pixels, several exposures were used for different samples. Comparisons of the absolute integrated density values for each line were made using the same exposure. For more information, see the Materials and methods. (F) Densitometry of the GFP–ATG8a cleavage assay in (E) confirms elevated autophagic flux in *ATG* OE backgrounds. The experiment was repeated twice; at least three western blot assays were performed for each experiment. The figure shows a representative example. Free GFP was expressed as a percentage of the total amount of GFP for each sample in (E). Data represent means ±SE of three individual measurements. The means of each genotype were compared with the WT using Dunnett’s test, ***P<0.001.

Next, we studied the effect of enhanced Atg8 lipidation in *ATG* OE lines on autophagic flux by analyzing the efficacy of autophagosome formation (Fig. 2A, B; Supplementary Fig. S4) and degradation of potential autophagosomal cargo (Fig. 2C–F). Plants were treated with ConA to block vacuolar lysis and to cause accumulation of undegraded GFP–Atg8a-labeled autophagic bodies in the vacuolar lumen. We found an increased number of GFP-positive puncta in *ATG5* OE and *ATG7* OE lines co-expressing the GFP–Atg8a marker (Fig. 2A, B). Interestingly, assessment of the area of GFP-positive puncta did not reveal any difference in their size in the overexpressing lines compared with the WT (Supplementary Fig. S4), indicating that overexpression of *ATG5* or *ATG7* increases only the number of autophagosomes, but not their size.

Degradation of the autophagic adaptor protein NBR1 (Svenning *et al.*, 2011; Minina *et al.*, 2013*b*; Klionsky *et al.*, 2016) as well as the accumulation of free GFP in GFP–Atg8-expressing cells (Nair *et al.*, 2011) are indicative of the completion of autophagic flux. Detection of NBR1 protein (Fig. 2C, D, top chart) was combined with the quantification of *NBR1* mRNA in the same leaf samples (Fig. 2D, bottom chart) to exclude the possibility that observed differences in NBR1 abundance were caused by variation at the transcriptional level. Analysis of GFP–Atg8a cleavage leading to accumulation of free GFP was performed on seedlings grown under normal conditions or subjected to starvation (Fig. 2E, F). The assay revealed increased autophagic flux in *ATG5* OE and *ATG7* OE lines. Therefore, we conclude that Arabidopsis lines overexpressing either *ATG5* or *ATG7* might represent a potent tool for studying the impact of enhanced autophagy on plant development and physiology.

### Enhanced autophagy promotes plant growth and suppresses aging

**Fig. 3.**
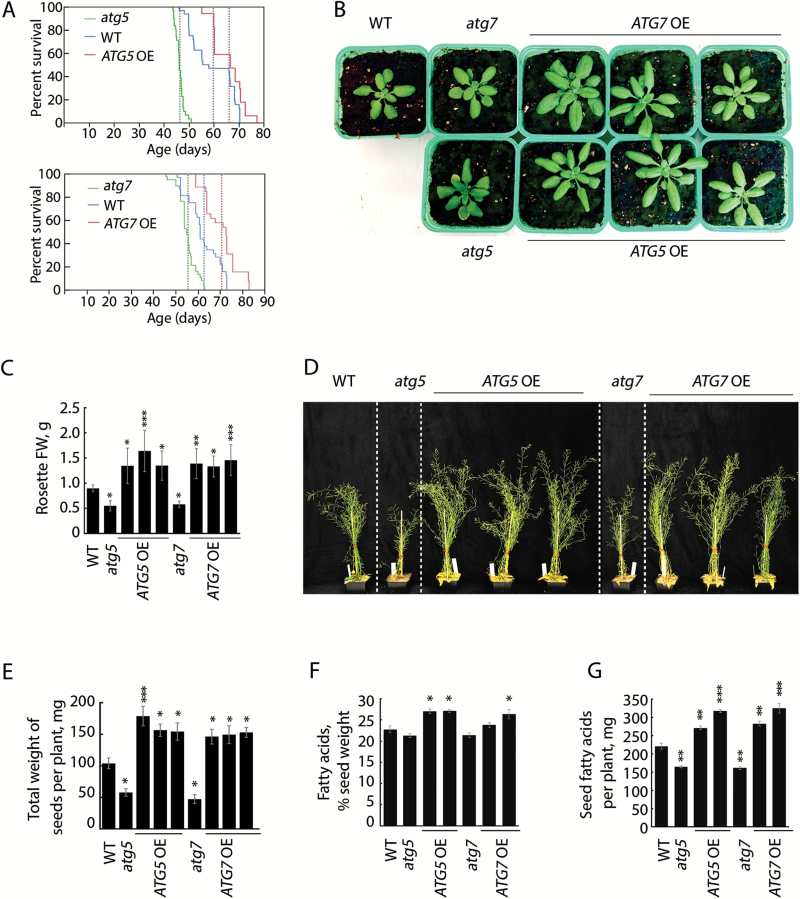
Enhanced autophagy extends plant life span and promotes vegetative growth, seed production, and accumulation of seed oil. (A) Kaplan–Meier survival curves for the WT plants, *ATG*-knockout (*atg5* and *atg7*), and *ATG*-overexpressing lines (*ATG5* OE and *ATG7* OE) grown under normal conditions (150 µE m^–2^ s^–1^ light, 16 h photoperiod). The dashed lines show mean life spans for different genotypes. Each of the two trials was repeated twice, every time with a different *ATG5*- or *ATG7*-overexpressing line. The life span of an individual plant was measured as the time period from radicle emergence to complete senescence of the rosette and cessation of flowering. (B) Representative phenotype of 3-week-old plants of the WT, *ATG*-knockout mutants, and three individual *ATG5*- or *ATG7*-overexpressing lines grown under normal conditions . Planting pot size was 8 × 8 cm. (C) Fresh weight of rosettes. Data represent means ±SE, *n*=3–4. ***P<0.0001; **P<0.001; *P<0.05; versus control (WT), Dunnett’s test. (D) Representative phenotype of plants of the same genotypes as in (B) at the flowering stage. Planting pot size was 8 × 8×8 cm. (E) The seed yield of WT, *ATG*-knockout, and *ATG*-overexpressing plants grown under normal conditions. Data represent means ±SE, *n*=6–11. ***P<0.0001; **P<0.001; *P<0.05; versus the WT, Dunnett’s test. (F, G) Oil content of seeds expressed as a percentage of seed weight (F) and in mg per plant, normalized to the seed yield presented in E (G). Acyl groups were measured in mature seeds harvested from three plants per genotype grown under normal conditions . Data represent means ±SE, *n*=3. ***P<0.001; **P<0.01; *P<0.05; versus the WT, Dunnett’s test.

Accelerated senescence of rosette leaves is a phenotypic hallmark of Arabidopsis *atg* mutants (Doelling *et al.*, 2002; Hanaoka *et al.*, 2002). Accordingly, we observed earlier onset and a more rapid progression of leaf senescence in the *atg5* and *atg7* backgrounds (Table 1). In contrast, plants from both *ATG5* OE and *ATG7* OE lines exhibited a significantly delayed onset (by 4–7 d) of leaf senescence, as compared with WT plants, albeit the duration of leaf senescence was not affected (Table 1). Notably, while the onset of flowering was independent of the level of autophagy, the duration of the flowering period correlated with autophagic flux, so that overexpressors of *ATG5* or *ATG7* flowered for ~10 d longer than WT plants (Table 2). As a result, plants of different overexpressor lines had on average a 10–20% longer life span compared with WT plants (Fig. 3A).

**Table 1.** Enhanced autophagy delays onset of leaf senescence

Genotype	Onset of rosette senescence, DAG	Complete rosette senescence, DAG	Duration of rosette senescence, d
*ATG5* trial			
WT	36.1 ± 3.74	55.6 ± 5.13	19.5 ± 5.08
*atg5*	30.2 ± 1.86****	44.5 ± 2.13****	14.3 ± 2.58****
*ATG5* OE	40.1 ± 3.55**	58.6 ± 3.52*	18.5 ± 5.22 ns
*ATG7* trial			
WT	35.1 ± 5.11	56.2 ± 5.00	21.1 ± 3.50
*atg7*	31.2 ± 4.91**	47.6 ± 5.39****	16.4 ± 4.03****
*ATG7* OE	42.1 ± 5.38**	62.1 ± 2.53**	20.0 ± 5.10 ns

DAG, days after germination (radicle emergence); OE, overexpression.

All time data are shown as the mean ±SD, with 20 plants per genotype.

Each trial was repeated twice, every time with a different overexpression line. Although there was variation among replicate trials in the absolute mean values, they all showed the same effects. *P<0.05, **P<0.01, ***P<0.001, ****P<0.0001; ns, not significant versus the WT in the same trial; Dunnett’s test.

**Table 2.** Enhanced autophagy sustains flowering

Genotype	First flower open, DAG	Cessation of flowering, DAG	Duration of flowering, d
*ATG5* trial			
WT	27.5 ± 1.61	58.7 ± 9.61	31.2 ± 9.89
*atg5*	27.8 ± 1.75 ns	46.4 ± 1.72****	18.6 ± 1.93****
*ATG5* OE	27.4 ± 1.37 ns	67.6 ± 5.99*	40.2 ± 7.05*
*ATG7* trial			
WT	30.0 ± 4.36	62.5 ± 7.35	32.6 ± 6.19
*atg7*	28.5 ± 4.02 ns	55.3 ± 4.04****	26.9 ± 4.77****
*ATG7* OE	32.4 ± 3.28 ns	74.3 ± 3.25****	41.9 ± 3.54****

DAG, days after germination (radicle emergence); OE, overexpression. All time data are shown as the mean ±SD, with 20 plants per genotype.

Each trial was repeated twice, every time with a different overexpression line. Although there was variation among replicate trials in the absolute mean values, they all showed the same effects. *P<0.05, **P<0.01, ***P<0.001, ****P<0.0001; ns, not significant versus the WT in the same trial; Dunnett’s test.

Apart from accelerated aging, autophagy-deficient plants exhibit reduced fecundity and suppressed vegetative growth, even when grown under nutrient-rich conditions (Hanaoka *et al.*, 2002; Bassham *et al.*, 2006; Guiboileau *et al.*, 2012, 2013). Accordingly, *atg5* and *atg7* plants grown under standard growth conditions showed an 50% reduction in both rosette fresh weight (Fig. 3B, C; Supplementary Fig. S5A) and the total weight of mature seeds per plant (Fig. 3E). In contrast, *ATG* overexpressors exhibited increased vegetative growth and seed yield (Fig. 3B, C, E; Supplementary Fig. S5A), although the weight of an individual seed was not affected (Supplementary Fig. S6). Therefore the observed variation in seed yield was caused by differences in fecundity, eventually reflecting a strong impact of the basal autophagy activity on flowering duration (Table 2). *ATG5*- or *ATG7*-overexpressing plants developed more and taller inflorescences than the respective knockout mutants and WT plants (Fig. 3D), flowered longer (Tables 1, 2), and thus produced more seeds per plant (Fig. 3E).

Importantly, the observed phenotypes were dependent on the growth conditions. The phenotypic differences between WT and *ATG*-overexpressing plants were not significant under autophagy-stimulating conditions, namely under light intensity level reduced to 100 μE m^–2^ s^–1^ (data not shown; Minina *et al.*, 2013*b*).

Interestingly, tracking of rosette development revealed a trend in *ATG*-overexpressing lines to develop more leaves compared with WT plants, whereas *ATG*-knockout plants developed a slightly lower number of leaves (Supplementary Fig. S5A). However, the observed differences in leaf number between WT and *ATG*-overexpressing plants were not statistically significant at any developmental stage and therefore did not influence the onset of flowering (Table 2).

The increased biomass (Fig. 3C) and larger size of leaf rosettes (Fig. 3B; Supplementary Fig. S5A) of *ATG*-overexpressing plants and opposite traits in the *ATG*-knockouts point to importance of autophagy in leaf cell expansion and/or division. Analysis of the cell size in fully expanded rosette leaf blades did not reveal any statistically significant difference between *ATG* overexpressors, WT, or *ATG*-knockouts (Supplementary Fig. S5B), suggesting that autophagy facilitates leaf cell division.

It was shown that autophagy can regulate fatty acid and lipid metabolism in animals through the recycling of lipid droplets in various cell types (in the process termed lipophagy; Singh *et al.*, 2009). Furthermore, autophagy mediates differentiation of white adipocytes, a cell type specialized in the storage of large unilocular lipid droplets (Zhang *et al.*, 2009).

Arabidopsis accumulates massive amounts of fatty acids in the form of triacylglycerols in seeds, making it a robust model for studying oil biosynthesis pathways and translating these findings to oil-seed crops (Bates *et al.*, 2013). We found that autophagy stimulates seed lipid accumulation, since knockout and overexpression of *ATG5* or *ATG7* led to a moderate decrease and increase of fatty acid content, respectively, compared with the WT (Fig. 3F). Since enhanced autophagy boosts seed set (Fig. 3E), the stimulatory effect of enhanced autophagy on seed oil yield per plant was much stronger, in the range of a 25–50% increase compared with the WT (Fig. 3G). Interestingly, the *atg5* and *atg7* knockouts had a significant change in seed fatty acid composition compared with the WT; the oleic acid (18:1) proportion was decreased with a corresponding increase in eicosa-13,16-dienoic acid (20:2) and erucic acid (22:1) (Supplementary Fig. S7), whereas *ATG*-overexpressing plants did not differ from the WT in this respect.

Collectively, our data demonstrate that Arabidopsis plants with constitutively enhanced autophagic flux show an increased vigor: longevity, vegetative growth, and fecundity. Additionally, we show the positive effect of constitutively up-regulated autophagy on seed oil accumulation.

### Enhanced autophagy confers increased resistance to necrotrophic pathogens and oxidative stress

Reallocation of resources from growth to stress resistance (and vice versa) determines the fitness costs, and their cutbacks represent an important task in plant breeding and biotechnology (Brown, 2002; Cabello *et al.*, 2014). Since autophagy plays a pivotal role in energy reallocation and metabolism and, as shown in this study, plants with enhanced autophagy gain in growth fitness, we hypothesized that these plants might ultimately lose in stress resistance.

**Fig. 4.**
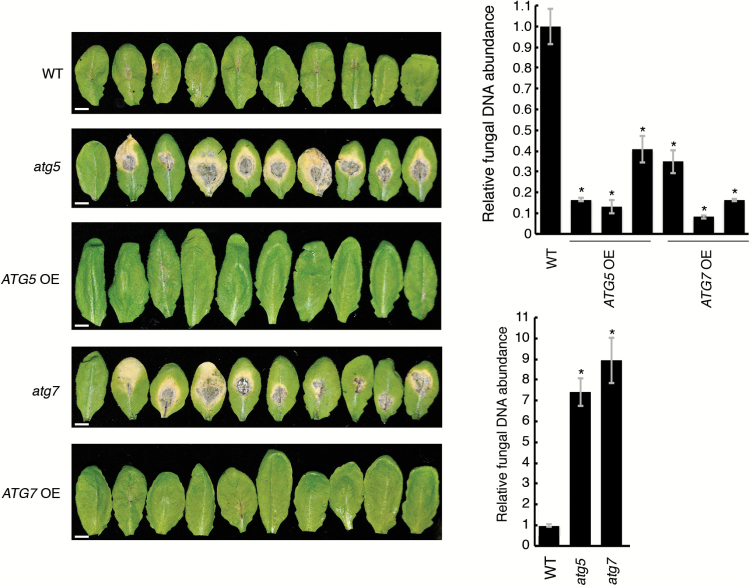
Plants with enhanced autophagy are more resistant to necrotrophic phytopathogens. Three-week-old plants of the WT, *ATG*-knockout, and three individual *ATG*-overexpressing lines were inoculated with 10 µl of suspension containing 5 × 10^5^ spores ml^–1^ of *Alternaria brassicicola*. Images display all rosette leaves from representative selected plants on the seventh day post-inoculation. Charts display fungal growth assessed by measuring fungal DNA using qRT-PCR to detect the fungal cutinase gene. Data represent means ±SE normalized to two reference genes (*UBQ5* and *PR2*), *n*≥3. *P<0.0001; versus the WT, Dunnett’s test. Scale bars=1 cm.

Previous observations revealed increased fungal growth in Arabidopsis *atg* mutants, indicating that autophagy is required for plant resistance to necrotrophic fungi (Lai *et al.*, 2011; Lenz *et al.*, 2011). In agreement with these studies, *atg5* and *atg7* plants developed unrestricted necrotic leaf lesions following inoculation with *A. brassicicola*, thereby greatly facilitating fungal growth (Fig. 4). In contrast, *ATG5*- or *ATG7*-overexpressing plants developed fewer necrotic lesions and showed suppressed fungal growth compared with WT plants (Fig. 4).

**Fig. 5.**
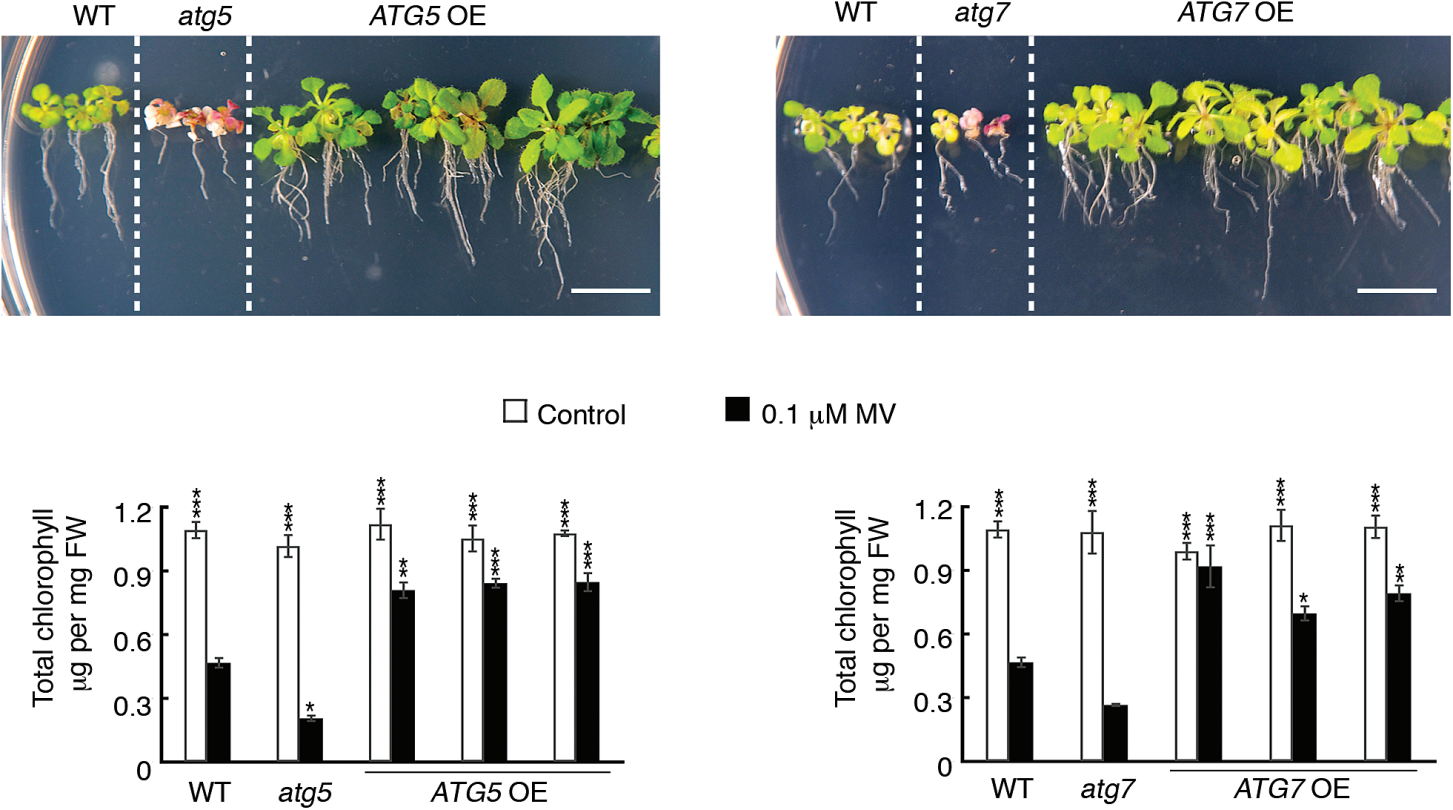
Plants with enhanced autophagy are more resistant to oxidative stress. Seeds of the WT, *ATG*-knockout mutants, and three individual *ATG5*- and *ATG7*-overexpressing lines were germinated on MS plates with or without addition of 0.1 µM methyl viologen (MV). The phenotype was assessed after 3 weeks. The difference in the tolerance to oxidative stress between different genotypes was confirmed by measuring chlorophyll content. Untreated plants (control) of all genotypes exhibited no significant difference in the chlorophyll content. Upon treatment with MV, WT and *ATG*-knockout plants showed a dramatic decrease in the chlorophyll content, while *ATG*-overexpressing lines maintained high levels of chlorophyll. Data represent means ±SEM, *n*=3–7. ***P<0.0001; **P<0.001; *P<0.05; versus MV-treated WT, Dunnett’s test. Scale bars=1 cm.

We reasoned that if enhanced autophagy plays a cytoprotective role during the necrotrophic infection, it might also aid in decreasing plant susceptibility to oxidative stress, which represents one of the major components of necrotrophic pathogenicity (AbuQamar *et al.*, 2006; Choquer *et al.*, 2007). In our experiments, *atg5* and *atg7* plants exhibited stronger growth suppression and chlorosis than WT plants when exposed to oxidative stress induced by MV treatment (Fig. 5). Correspondingly, *ATG*-overexpressing lines demonstrated enhanced resistance to MV, with their chlorophyll content levels slightly below the corresponding control (untreated plants) and much higher than those of MV-treated WT plants (Fig. 5).

Taken together, these data imply that elevated autophagy can improve plant resistance to both necrotrophic pathogens and oxidative stress.

### Transcriptional profiling of plants with enhanced autophagy

To investigate further molecular mechanisms underlying improved growth and stress resistance phenotypes of the *ATG*-overexpressing plants, we performed transcriptome analyses of rosette leaves at two time points representing distinct developmental stages. For the first time point, complete rosettes were sampled at the budding stage, when no difference in phenotype of WT, *ATG*-knockout, and *ATG*-overexpressing plants was detectable. The second sampling was performed 10 DAF, when *ATG*-knockout plants showed early signs of senescence, and differences between WT and *ATG*-overexpressing plants became detectable at the molecular level (Fig. 2C, D).

Expression of each transcript at either time point was first normalized to the corresponding values in the WT. Next, transcripts were sorted to select only those that displayed common expression trends in both *atg5* and *atg7* mutants or in both *ATG5* OE and *ATG7* OE lines.

Our results confirm general transcriptional trends of Arabidopsis *atg* mutants reported previously (Masclaux-Daubresse *et al.*, 2014; Avin-Wittenberg *et al.*, 2015) and also indicate the presence of a complex signaling similar to the immune response, induction of pathways managing oxidative stress, and elevated response to salicylic acid (Supplementary Tables S2–S4). We did not observe the previously reported up-regulation of methionine and ethylene biosynthesis (Masclaux-Daubresse *et al.*, 2014) either in *atg5* or in *atg7* plants, which might be explained by the differences in sampling stages.

**Fig. 6.**
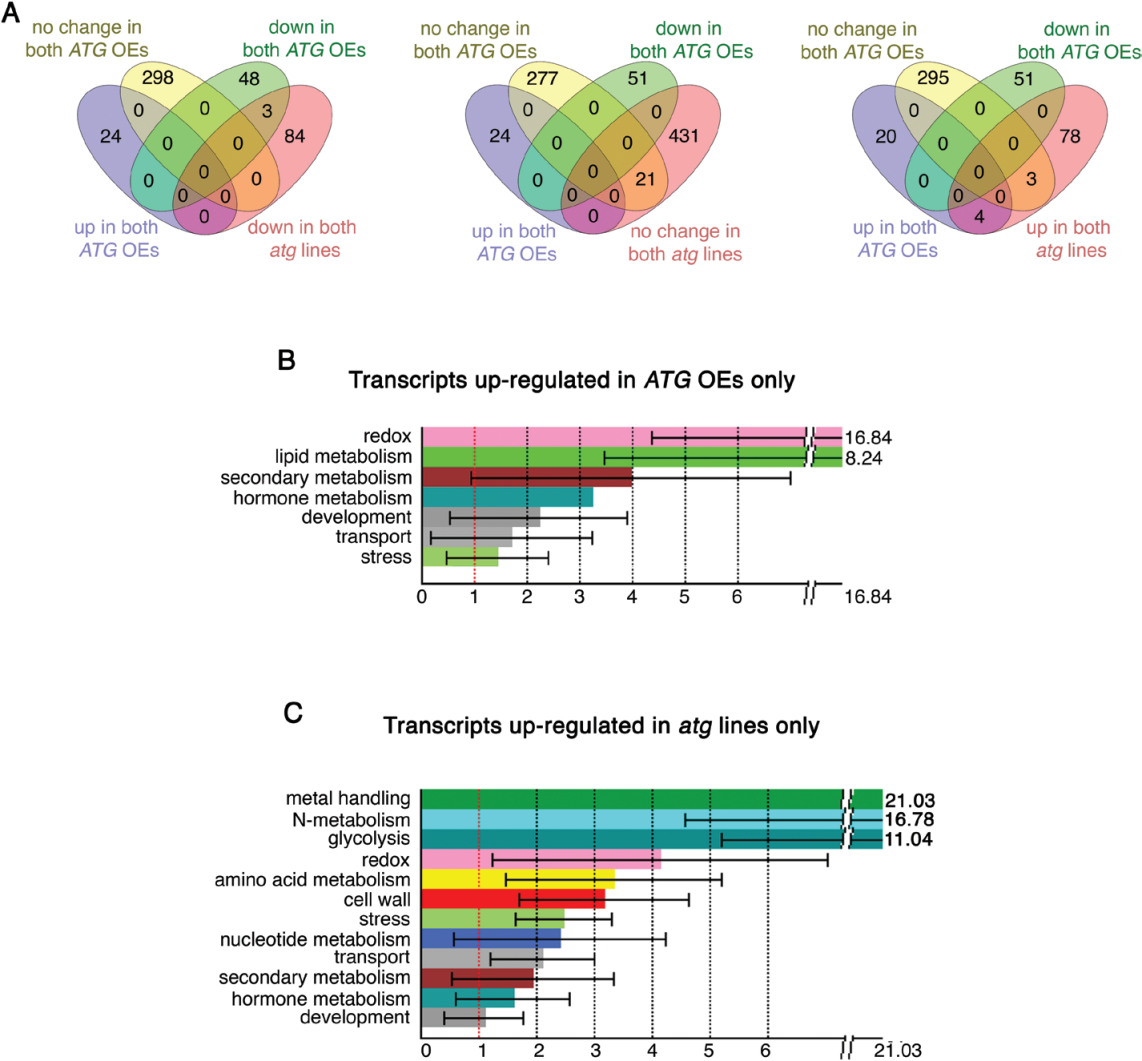
Transcriptional trends characteristic for *ATG*-overexpressing and *ATG*-knockout lines at the bolting stage. Complete rosettes from four individual plants of each genotype were harvested at the bolting stage (0 DAF) and pooled together for RNA extraction followed by microarray analysis. Differentially expressed genes (DEGs) were selected by normalizing values obtained for transgenic backgrounds to the corresponding expression values obtained for the WT background. Only the genes showing common trends for both *ATG5*- and *ATG7*-overexpressing backgrounds (*ATG* OEs) or both *ATG5*- and *ATG7*-knockout backgrounds (*atg* lines) were used for further analysis. (A) Venn diagrams illustrating the number of DEGs (genes expressed differently from the WT at the bolting stage) in *ATG*-overexpressing and *ATG*-knockout plants. (B, C) Gene ontology analysis of DEGs specific for *ATG*-overexpressing (B) or *ATG*-knockout (C) plants.

In agreement with the results of the phenotypic analysis, the number of differentially expressed genes at the first time point was relatively low (Fig. 6A; Supplementary Table S3). Nevertheless, already at this stage we could observe an increase in the expression of enzymes involved in lipid metabolism in *ATG*-overexpressing plants, while *atg* plants displayed up-regulation of stress- and starvation-related genes (Fig. 6B, C; Supplementary Table S3).

**Fig. 7.**
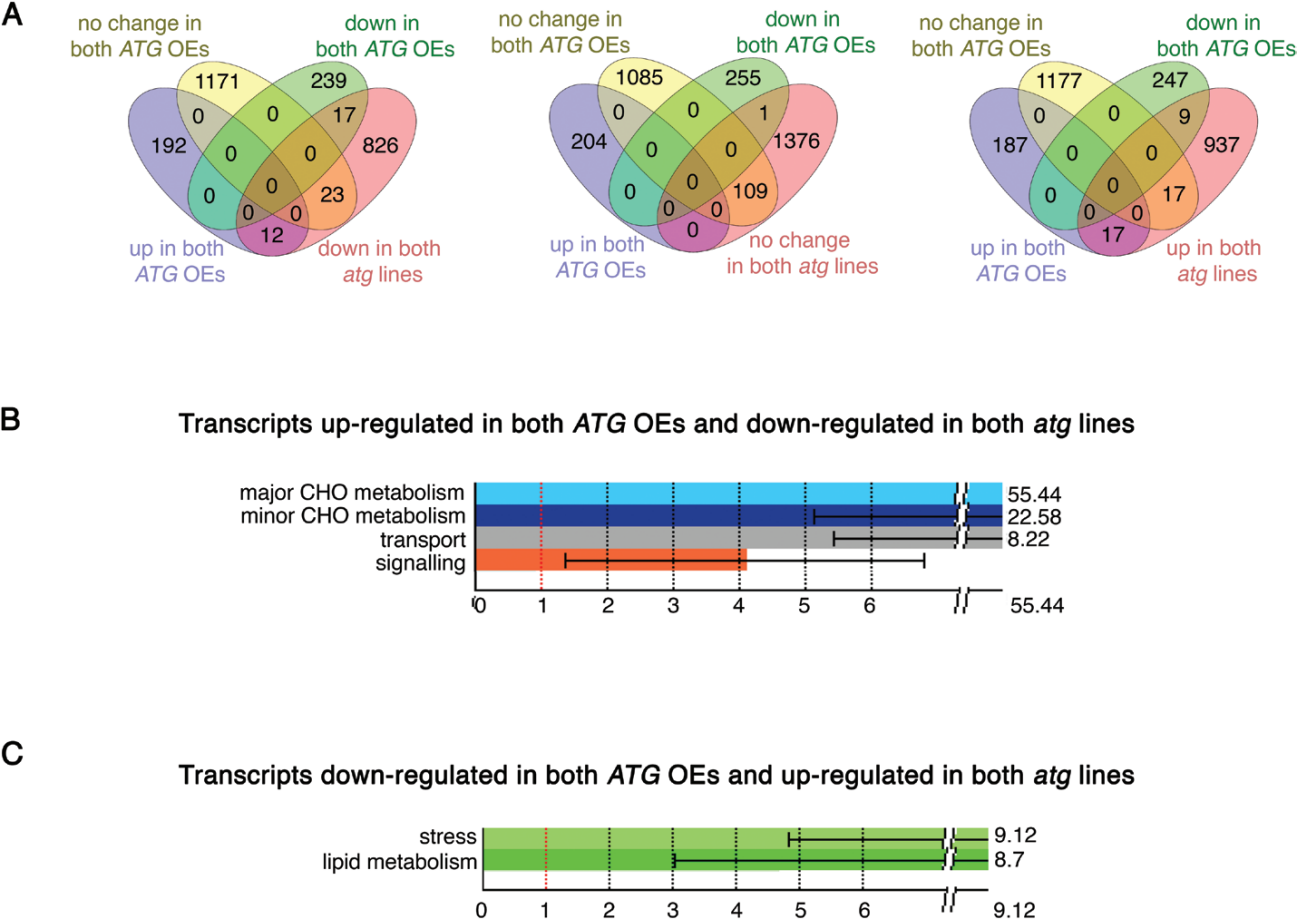
Transcriptional trends characteristic for *ATG*-overexpressing and *ATG*-knockout lines at the 10 DAF stage. Complete rosettes from four individual plants of each genotype were harvested at 10 DAF and pooled together for RNA extraction followed by microarray analysis. Differentially expressed genes (DEGs) were selected by normalizing values obtained for transgenic backgrounds to the corresponding expression values obtained for the WT background. Only the genes showing common trends for both *ATG5*- and *ATG7*-overexpressing backgrounds (*ATG* OEs) or both *ATG5*- and *ATG7*-knockout backgrounds (*atg* lines) were used for further analysis. (A) Venn diagrams illustrating the number of DEGs (genes expressed differently from the WT at 10 DAF) in *ATG*-overexpressing and *ATG*-knockout plants. (B, C) Gene Ontology analysis of DEGs showing opposite expression trends in *ATG*-overexpressing and *ATG*-knockout plants.

At the second time point, the number of DEGs significantly increased for both *atg* and *ATG*-overexpressing plants, and the opposite trends became readily identifiable (Fig. 7; Supplementary Tables S1, S4). In general, we could observe an increase in transcripts involved in proteolysis, lipid degradation, and salicylic acid signaling in *atg* plants and an opposite trend in *ATG*-overexpressing plants.

One of the causes of the early onset of senescence in *atg* plants was proposed to be their susceptibility to UV light and reactive oxygen species (ROS). This phenomenon has been linked to the decreased production of flavonoids and anthocyanin observed in *ATG*-deficient plants (Masclaux-Daubresse *et al.*, 2014). Interestingly, a large number of genes involved in flavonoid biosynthesis and anthocyanin production, as well as in oxidative stress response were up-regulated in *ATG*-overexpressing plants (Supplementary Tables S1, S4). Furthermore, at the developmental stages later than 10 DAF (second time point), *ATG*-overexpressing plants accumulated visibly higher amounts of anthocyanin than WT plants (data not shown), thus confirming the functionality of transcriptional up-regulation of the anthocyanin biosynthesis pathway. This observation is also in agreement with the recent reports proposing the link between elevated anthocyanin production and enhanced autophagy flux in plants overexpressing one of the apple orthologs of ATG18 (Sun *et al*., 2017; Sun *et al*., 2018).

It is noteworthy, that at the second time point, sugar transport genes were significantly down-regulated in *atg* plants and up-regulated in *ATG*-overexpressing plants when compared with the WT (Fig. 7; Tables S1, S2 and S4). Transport of sugars from rosette leaves to the inflorescence is essential for sustaining seed onset and development (Wingenter *et al.*, 2010). Thus, higher seed yield of *ATG*-overexpressing plants could be attributed to the combined effect of the long-lasting rosette and high efficacy of sugar transport towards the inflorescence.

## Discussion

### Transcriptional regulation of autophagy

Previous studies in animals and plants demonstrate that artificial manipulation of autophagy can drastically affect various aspects of organismal physiology related to growth, aging, and diseases (Fleming *et al.*, 2011; Rubinsztein *et al.*, 2011; Liu and Bassham, 2012). In plants, these studies were mainly conducted using *ATG*-knockout mutants and RNAi lines, which allowed compromised growth and stress resistance coupled with accelerated aging as a consequence of suppressed autophagy to be revealed. Here we present evidence that constitutive overexpression of *ATG5* or *ATG7* in Arabidopsis is sufficient to enhance lipidation of Atg8, leading to the formation of a higher number of autophagosomes, enhanced autophagic flux, and improved plant fitness.

**Fig. 8.**
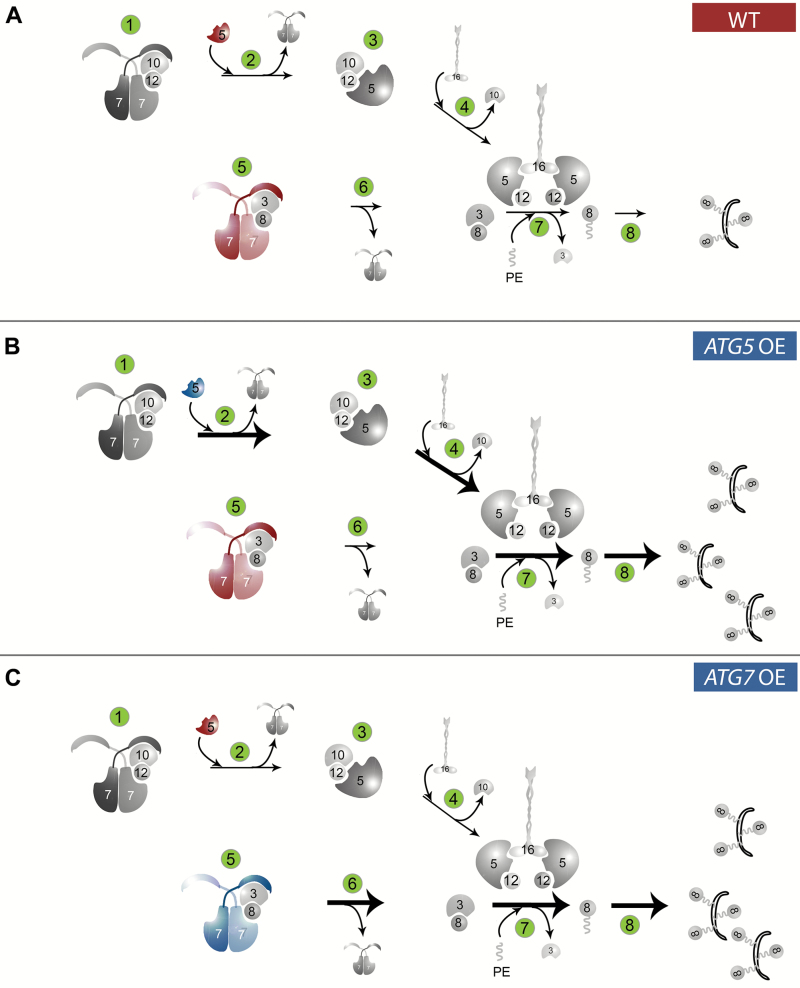
A schematic representation of the proposed effects of *ATG5* or *ATG7* overexpression on the efficacy of Atg8 lipidation. (A) A schematic representation of two autophagy-related ubiquitin-like conjugation systems in the WT background. Atg7 acts as an E1-like ligase catalyzing formation of Atg12–Atg10 (step 1) and Atg8–Atg3 (step 5) conjugates. Atg12 is further transferred onto Atg5 and the resulting Atg12–Atg5 conjugate forms a complex with Atg16 (steps 2–4). The Atg12–Atg5–Atg16 complex serves as an E3-like ligase for conjugating Atg8 with a phosphatidylethanolamine (PE) (steps 6 and 7). Lipidated Atg8 contributes to elongation of a phagophore (step 8). Red color indicates pools of Atg5 and Atg7 that act as rate-limiting components of the corresponding reactions. (B) Atg5 is a limiting component of steps 2–4, 7, and 8. Constitutive overproduction of Atg5 (blue color) in the *ATG5 OE* background does not influence expression profiles of other components of the conjugation systems but is sufficient to stimulate conjugation of Atg12 with Atg5 and of Atg8 with PE, indicating enhanced efficacy of steps 2–4, 7, and 8 (bold arrows). (C) Atg7 is a limiting component of steps 5–8. Overexpression of *ATG7* (blue color) also does not change expression of other *ATG* genes involved in the ubiquitin-like conjugation systems. In agreement with the assumption that Atg5 is the limiting component of steps 2 and 3 of the pathway, the Atg12–Atg5 conjugate does not accumulate in *ATG7* OE plants. However, *ATG7* OE plants still have a higher amount of the Atg8–PE conjugate, suggesting enhanced efficacy of steps 7 and 8 (bold arrows). This indicates that the constitutively enhanced abundance of Atg7 (blue color) boosts steps 5 and 6 (bold arrow). Possibly, increased formation of the Atg8–Atg3 conjugate stimulates Atg8 lipidation without requiring more of the Atg12–Atg5–Atg16 complex. Enhanced lipidation of Atg8 in *ATG5* OE or *ATG7* OE plants does not lead to changes in the size of autophagosomes, but instead augments the frequency of phagophore formation, as illustrated in (B) and (C).

Regulation of autophagy is complex and includes transcriptional, post-transcriptional, and post-translational steps (Feng *et al.*, 2015). One of the post-translational modifications essential for the formation of autophagosomes is lipidation of Atg8 mediated by two ubiquitin-like conjugation systems (Fig. 8A). Here we demonstrate that Atg5 is a rate-limiting factor in the Atg12–Atg5 conjugation pathway, and accumulation of a higher amount of the Atg12–Atg5 conjugate upon overexpression of *ATG5* tightly correlates with the efficacy of Atg8–PE formation (Fig. 8B). Furthermore, we show that although overexpression of *ATG7* does not contribute to Atg12–Atg5 conjugation, it still boosts lipidation of Atg8, suggesting that higher efficacy of Atg8–Atg3 conjugation is beneficial for the Atg8 lipidation rate even at the ‘normal’ abundance of the Atg12–Atg5–Atg16 complex (Fig. 8C). Consequently, we suggest that Atg8 lipidation can be enhanced either by increasing amounts of the E3-like ligase (Atg12–Atg5–Atg16 complex) or by boosting Atg5-unrelated activity of Atg7, for example formation of the intermediate conjugate Atg8–Atg3. Whether artificial transcriptional up-regulation of other *ATG* genes can be instrumental in controlling the autophagic flux or will be just a bystander event (as in the case of Arabidopsis *ATG8a*) remains an open question.

Importantly, while overexpression of *ATG* genes in yeast does not significantly affect autophagic activity (Ma *et al.*, 2007), several studies, including this work, demonstrate that overexpression of some of these genes in plant and animal models has a stimulatory effect on autophagic flux (Scott *et al.*, 2007; Pyo *et al.*, 2013). A plausible explanation for these contrasting observations made in yeast and other organisms could be the differences between the number of phagophore assembly sites or pre-autophagosomal structures (PASs) at which the core Atg proteins function. While yeast cells possess only a single PAS, plant and animal cells do not seem to have any limitation in the potential number of PASs per cell (Parzych and Klionsky, 2014). Thus, overexpression of rate-limiting PAS components might increase the number of forming PASs and result in the higher frequency of autophagosome formation, as was indeed observed in this study.

In plants, transient overexpression of *ATG3* in *Nicotiana benthamiana* (Han *et al.*, 2015) or stable overexpression of either *ATG5* or *ATG7* in Arabidopsis (present study) is sufficient to stimulate autophagy. In contrat, overexpression of *ATG8* genes fused to a fluorescent protein, which is routinely used for live imaging of autophagosomes (Bassham, 2015; Klionsky *et al.*, 2016), has never been reported to enhance autophagy. An increased vegetative growth and seed production previously observed upon heterologous expression of soybean *ATG8c* in Arabidopsis (Xia *et al.*, 2012) might be caused by autophagy-independent functions of the Atg8c protein. A recent study of heterologous overexpression of apple *ATG7* in Arabidopsis plants (Wang *et al.*, 2016) reports some observations similar to those demonstrated in this study, such as enhanced biomass, but also some phenotypes not found in the Arabidopsis lines overexpressing Arabidopsis *ATG7*, such as accelerated bolting. This finding indicates that heterologous Atg proteins might bring some additional functions besides their participation in autophagy and/or different efficacy when compared with the native homologs.

Interestingly, while individual overexpression of either *ATG5* or *ATG7* did not influence expression of other components of the ubiquitin-like conjugation systems (Supplementary Figs S2, Fig. S3C), simultaneous overexpression of both genes led to a significant suppression of other *ATG* genes involved in the conjugation systems, showing trends opposite to expression profiles observed in the *ATG*-knockout plants (Fig. S3C). These results indicate the presence of a complex feedback loop controlling autophagic activity at the transcriptional level. While a number of transcription factors and other regulatory proteins directly targeting *ATG* genes have been identified in animal models (Lee *et al.*, 2014; Seok *et al.*, 2014; Feng *et al.*, 2015; Lapierre *et al.*, 2015), master transcription regulators of plant autophagy remain unknown.

### Phenotypic expression of manipulated autophagy

Autophagy plays a multifaceted role in plant physiology by performing a broad array of cellular and organismal functions. Therefore, it is not surprising that enhancement of the autophagic flux in Arabidopsis by overexpressing *ATG5* or *ATG7* affected so many traits, ranging from longevity to lipid biosynthesis and stress resistance.

A potential reason for the delayed leaf senescence, as well as for increased vegetative growth of *ATG5*- or *ATG7*-overexpressing plants (Fig. 3B, C; Table 1; Supplementary Fig. S5) might be linked to the pathway of starch remobilization, which is reliant on the autophagy-dependent targeting of non-plastidial starch granule-like structures to the vacuoles for degradation (Wang *et al.*, 2013). Autophagy was also shown to be necessary for delaying senescence by suppressing salicylic acid signaling (Yoshimoto *et al.*, 2009), and our results indicate that this pathway might contribute to the longevity phenotype of the plants with constitutively up-regulated autophagy (Fig. 3A; Table 1; Supplementary Table S1). Additionally, autophagy participates in the recycling of chloroplastic proteins and whole chloroplasts in leaves (Ishida *et al.*, 2008; Wada *et al.*, 2009), thus supporting nitrogen remobilization and use efficiency (Guiboileau *et al.*, 2012, 2013; Ren *et al.*, 2014; Li *et al.*, 2015; Wada *et al.*, 2015). The more efficient flux of nitrogen from source to sink might result in a better support of apical shoot meristem and thus more flowers and increased seed set, both traits consistently observed in the transgenic plants with enhanced autophagy (Table 2; Fig. 3D, E).

Studies in animal systems revealed a complex crosstalk between lipids and autophagy. Not only are numerous lipids, free fatty acids, and enzymes of lipid metabolism involved in modulation (usually stimulation) of autophagy, but autophagy in return controls the lipid status of the cell, tissue, and whole organism through a process of selective recycling of lipid droplets (lipophagy) and by an as yet undefined molecular mechanism conferring differentiation of adipose tissues (Dall’Armi *et al.*, 2013; Liu and Czaja, 2013). It remains unknown whether a similar or distinct mechanistic connection between autophagy and lipid metabolism exists in plants (for a review, see Elander *et al*, 2018). Recent lipidomics analysis of *atg5* and *atg7* Arabidopsis seedlings subjected to carbon deprivation revealed increased accumulation of most classes of fatty acids in *atg5* mutants and most classes of lipids in both *atg5* and *atg7* mutants, indicating that autophagy is required for lipid catabolism during seedling growth under carbon-deprived conditions (Avin-Wittenberg *et al.*, 2015). It is not feasible to compare these results with results obtained in our study due to major differences in the experimental design, namely seedlings grown under starvation versus seeds maturing under nutrient-rich conditions. We observed a direct correlation between the level of autophagy and seed fatty acid content (Fig. 3F, G), suggesting that increased autophagic flux mediates deposition of seed lipids. Both *atg5* and *atg7* mutants had similar changes in seed fatty acid composition, with a decrease in oleic acid and an increase in very long chain fatty acids, whereas the *ATG* overexpressors had a WT-like fatty acid composition (Supplementary Fig. S7). Thus, functional autophagy not only regulates the accumulation of lipids but can, at least to some extent, also regulate the fatty acid composition of these lipids.

Autophagy is a major cytoprotective mechanism activated during various stress responses to remove or recycle toxic compounds, protein aggregates, and defective organelles (Liu and Bassham, 2012). These homeostatic functions were proposed to underlie altered cell death and resistance phenotypes of autophagy-deficient mutants in comparison with the WT during pathogen infection. While the response of autophagy-deficient mutants to biotrophic pathogens appears to be variable due to age-dependent changes in the salicylic acid content and downstream signaling (Patel and Dinesh-Kumar, 2008; Hofius *et al.*, 2009; Yoshimoto *et al.*, 2009; Lenz *et al.*, 2011; Minina *et al.*, 2014; Zhou *et al.*, 2014), their enhanced susceptibility towards different necrotrophic pathogens has been consistently observed (Lai *et al.*, 2011) (Fig. 4). Our finding that *ATG5* and *ATG7* OE lines are more resistant to *A. brassicicola* further suggests an important and direct role for autophagy in plant immunity to necrotrophs. Such a contribution might be related to the suppression of a disease-associated necrotic cell death through removal of plant- or pathogen-derived toxic cellular constituents. Alternatively, autophagy is known to modulate jasmonic acid-dependent signaling positively (Lai *et al.*, 2011), which is an essential component of the immune system against necrotrophic pathogens. The recent observation that the fungus *Sclerotinia sclerotiorum* enhances virulence via phytotoxin-mediated suppression of autophagy further highlights the importance of autophagic mechanisms in the host defense against necrotrophs (Kabbage *et al.*, 2013).

Plants with enhanced autophagy are also more resistant to oxidative stress (Fig. 5) and show an elevated level of expression of genes involved in UV and oxidative stress responses, and anthocyanin and flavonoid biosynthesis (Supplementary Tables S1, S2). These data are in good agreement with the previously reported roles of autophagy in suppressing oxidative damage through clearance of oxidized proteins (Xiong *et al.*, 2007), protein aggregates (Zhou *et al.*, 2014), and defective peroxisomes (Shibata *et al.*, 2013) and with the most recent report suggesting a link between elevated anthocyanin production, clearance of the oxidized proteins and, possibly, enhanced autophagy in the plants overexpressing an apple ortholog of ATG18 (Sun *et al*., 2017). Further work is required to conclude how general is an improved disease and stress resistance of plants with an elevated level of basal autophagy by studying their responses to other types of pathogens and stresses.

It is important to emphasize that the observed plant phenotypes were highly dependent on growth conditions and were most prominent under standard conditions (16 h light, 150 µE m^–2^ s^–1^ light intensity, 22 °C, 75% humidity). Phenotypic differences between WT and *ATG*-overexpressing plants greatly diminished under conditions previously shown to induce autophagy in WT plants, namely under light intensity reduced to 100 µE m^–2^ s^–1^ (Minina *et al.*, 2013*b*; unpublished data), suggesting the existence of a wide range within which autophagic activity can be tuned by changing growth conditions.

### Concluding remarks

An important question is why do plants not have a constitutively up-regulated autophagy, considering its potential benefits. The high plasticity of autophagic activity and its condition-dependent tuning in WT plants indicate that under highly variable conditions constitutively up-regulated autophagy might be less beneficial than tunable autophagy. For instance, an extended life span might be a drawback in the field environment, exposing plants to suboptimal climate conditions and another set of pathogens. As of yet, we have not been able to identify any fitness costs of constitutively up-regulated autophagy. Further experiments in field conditions will allow us to discover the costs, if they exist.

In conclusion, our work revises an experimental paradigm of autophagy in plant biology by complementing data obtained using *atg* mutants with data obtained using plants with a constitutively elevated level of autophagy due to overexpression of *ATG5* or *ATG7*. In contrast to perturbed growth, decreased fecundity, and compromised stress resistance caused by the autophagy deficiency, elevated autophagy results in delayed aging, enhanced vegetative growth and seed production, increased accumulation of seed lipids, and improved resistance to necrotrophs and oxidative stress; that is, a significant improvement of a number of agronomically important traits. These results obtained in Arabidopsis are reminiscent of the reported anti-aging phenotype of *ATG5*-overexpressing mice, which displayed a longer life span, leanness, increased insulin sensitivity, improved motor function, and oxidative stress resistance (Pyo *et al.*, 2013). Taken together, these results demonstrate a cross-kingdom conservation of the pleiotropic invigorating effect of enhanced autophagy.

## Supplementary data

Supplementary data are available at *JXB* online.

Fig. S1. qRT-PCR analysis of *ATG5* and *ATG7* transcripts in WT and *ATG*-overexpressing plants.

Fig. S2. Overexpression of either *ATG5* or *ATG7* does not influence transcription of other components of the ubiquitin-like conjugation systems.

Fig. S3. Simultaneous overexpression of *ATG5* and *ATG7* has the same effect on Atg5–Atg12 conjugation as overexpression of *ATG5* only and causes transcriptional suppression of other components of the two ubiquitin-like conjugation systems.

Fig. S4. Size of GFP-positive puncta measured in the WT, *ATG*-overexpressing, and *ATG*-knockout backgrounds expressing GFP–Atg8a.

Fig. S5. Overexpression of *ATG5* or *ATG7* does not influence the number of rosette leaves or cell size.

Fig. S6. Overexpression of *ATG5* or *ATG7* does not influence the weight of an individual seed.

Fig. S7. Knockout but not overexpression of *ATG*5 or *ATG7* alters the composition of seed fatty acids.

Table S1. Selected differentially expressed genes.

Table S2. Developmental trends of gene expression common for both *atg5* and *atg7* mutants and both *ATG5* OE and *ATG7* OE lines.

Table S3. Expression profiles characteristic for both *atg5* and *atg7* mutants and both *ATG5* OE and *ATG7* OE lines at the first time point.

Table S4. Expression profiles characteristic for both *atg5* and *atg7* mutants and both *ATG5* OE and *ATG7* OE lines at the second time point

Table S5. List of primers used in this study

File S1. Guide to genome-wide gene expression analysis.

Methods S1.

Supplementary Table S1

Supplementary Table S2

Supplementary Table S3

Supplementary Table S4

Supplementary Table S5

Supplementary Figures

Supplementary Data

Supplementary Methods

## Abbreviations:

ATG: autophagy-related gene
Atg: autophagy-related protein
ConA: concanamycin A
DAF: days after the first flower opened
DEG: differentially expressed gene
MV: methyl viologen
NBR1: neighbor of BRCA1
PAS: phagophore assembly site or pre-autophagosomal structure
PE: phosphatidylethanolamine
ROS: reactive oxygen species
WT: wild type.
