# Supplementary material for: Transcriptional stimulation of rate-limiting components of the autophagic pathway improves plant fitness
Source: J Exp Bot. 2018 Jan 20;69(6):1415–32. doi: 10.1093/jxb/ery010 (PMC6019011; doi:10.1093/jxb/ery010)
Supplement: Supplementary Table S1 [file ery010_suppl_supplementary_table_s1.pdf]

Table S1. Selected differentially expressed genes.

Predicted function	ID	Gene name	Changes in expression		
			**WT**	**In both *atg* knockouts**	**In both *ATG* OE**
Cell expansion					
	AT2G20750	EXPANSIN B1	downregulated*	downregulated*	downregulated*
	AT2G37640	ATEXP3	downregulated*	downregulated*	downregulated*
	AT3G29030	ATEXP5	downregulated*	downregulated*	downregulated*
	AT3G55500	ATEXPA16	downregulated*	downregulated*	downregulated*
	AT1G26770	AT-EXP10	downregulated*	downregulated*	downregulated*
	AT2G28950	ATEXP6	downregulated*	downregulated*	downregulated*
	AT4G28250	EXPANSIN B3	downregulated*	downregulated*	downregulated*
	AT1G20190	ATEXP11	downregulated*	downregulated*	downregulated*
	AT3G03220	ATEXP13	downregulated*	downregulated*	downregulated*
	AT2G39700	ATEXP4	downregulated*	downregulated*	downregulated*
Response to UV-B[¶]					
	AT4G14690	ELIP2	N/A	no change**	upregulated**
	AT5G13930	CHALCONE SYNTHASE	N/A	no change**	upregulated**
	AT1G65060	4CL3	N/A	no change**	upregulated**
Anthocyanin biosynthesis[¶]					
	AT5G42800	DFR	N/A	no change**	upregulated**
	AT3G29590	AT5MAT	N/A	no change**	upregulated**
	AT1G56650	ATMYB75	N/A	no change**	upregulated**
	AT4G22880	ANTHOCYANIDIN SYNTHASE	N/A	no change**	upregulated**
Oxidative stress response[¶]					
	AT5G49330	ATMYB111	N/A	no change**	upregulated**
	AT3G62610	MYB11	N/A	no change**	upregulated**

	AT2G47460	ATMYB12	N/A	no change**	upregulated**
	AT3G51240	F3H	N/A	no change**	upregulated**
	AT4G34135	UGT73B2	N/A	no change**	upregulated**
	AT5G07990	CYP75B1	N/A	no change**	upregulated**
	AT3G55120	CFI, CHALCONE FLAVANONE ISOMERASE	N/A	no change**	upregulated**
	AT3G62960	Thioredoxin superfamily protein	N/A	no change**	upregulated**
	AT1G59730	THIOREDOXIN H-TYPE 7	N/A	no change**	upregulated**
	AT4G33040	Thioredoxin superfamily protein	N/A	no change**	upregulated**
	AT2G47880	Glutaredoxin family protein	N/A	no change**	upregulated**
	AT5G11930	Thioredoxin superfamily protein	N/A	no change**	upregulated**
Lipid storage					
	AT5G07530	ATGRP-7	N/A	no change**	downregulated**
	AT5G51210	OLEOSIN3	N/A	no change**	downregulated**
	AT5G07600	Oleosin family protein	N/A	no change**	downregulated**
	AT5G07560	GRP20	N/A	no change**	downregulated**
	AT5G07510	GRP14	N/A	no change**	downregulated**
	AT5G07550	GRP19	N/A	no change**	downregulated**
Proteolysis					
	At1g03230	Aspartyl protease family protein	N/A	upregulated**	downregulated**
	At1g03220	Aspartyl protease family protein	N/A	upregulated**	downregulated**
Sugar transport					
	At4g04750.1	Carbohydrate transmembrane transporter	N/A	downregulated**	upregulated**
	At4g04760.1	Carbohydrate transmembrane transporter	N/A	downregulated**	upregulated**
	At5g17700.1	Carbohydrate transmembrane transporter	N/A	downregulated**	upregulated**

Response to salicylic acid stimulus					
	AT1G48000	ATMYB112	N/A	no change***	downregulated***
	AT3G48920	ATMYB45	N/A	upregulated***	no change***
	AT3G56400	ATWRKY70	N/A	upregulated*	no change**
	AT5G54610	ANKYRIN	N/A	upregulated*	no change**
	AT5G22570	ATWRKY38	N/A	upregulated*	no change**

* Expression changes detected when comparing the 2^d time point to the 1st time point

** Expression changes detected when comparing pooled *atg* knockout or *ATG* OE to WT at the 2^d time point

*** Expression changes detected when comparing pooled *atg* knockout or *ATG* OE to WT at the 1st time point

¶ UV- and oxidative-stress responses, anthocyanin and flavonoid biosynthesis pathways are tightly interconnected, most of the genes participating in one of the pathways also contributes to the other three.

N/A, not applicable. For comparisons of expression trends normalized to WT first.
