# Supplementary material for: Transcriptional stimulation of rate-limiting components of the autophagic pathway improves plant fitness
Source: J Exp Bot. 2018 Jan 20;69(6):1415–32. doi: 10.1093/jxb/ery010 (PMC6019011; doi:10.1093/jxb/ery010)
Supplement: Supplementary Table S5 [file ery010_suppl_supplementary_table_s5.docx]

**Supplementary Table 5. List of primers used in this study.**

| **Primer name** | **5’ primer sequence 3’** |
| --- | --- |
| attB1-ATG5UTR-Fw | GGGGACAAGTTTGTACAAAAAAGCAGGCTATACAGAAACAGCGTCGTTTTG |
| attB2-ATG5-Rev | GGGGACCACTTTGTACAAGAAAGCTGGGTTCACCTTTGAGGAGCTTTCACAAG |
| AtATG5 qPCR Fw2 | ATACACTTTAGAGGATATCCTTGCA |
| AtATG5 qPCR Re2 | ACCGTTCATGACAGAGGTCCATA |
| AtATG7 qPCR Fw2 | TCTAATCCAGTCAGGCAATCTCT |
| AtATG7 qPCR Re2 | GATTCAATCAACTCGCTAAGGCGT |
| NBR1 qPCR Fw | GAGGACCCAGACCGGAAGG |
| NBR1 qPCR Re | gacaaacacgacgaggatgc |
| PP2A qPCR Fw | TAACGTGGCCAAAATGATGC |
| PP2A qPCR Re | GTTCTCCACAACCGCTTGGT |
| HEL qPCR Fw | CCATTCTACTTTTTGGCGGCT |
| HEL qPCR Re | TCAATGGTAACTGATCCACTCTGATG |
| FWatg7 | GGGGACAAGTTTGTACAAAAAAGCAGGCTTGATGGCTGAGAAAGAAACTCCA |
| RVatg7 | GGGGACCACTTTGTACAAGAAAGCTGGGTATTAAAGATCTACAGCTACATCG |
| UBQ 5 FW | GACGCTTCATCTCGTCC |
| UBQ 5 RV | CCACAGGTTGCGTTAG |
| PR2 FW | AGGAGCTTAGCCTCACCACC |
| PR2 RV | GAGGATGAGCTCGATGTCAGAG |
| Cutinase FW | ATCACTGCCGGTGGTTACTC |
| Cutinase FW | CGACACCCTTGATTTGGTCT |
| qPCR.AtATG10.Fw | AGGAAGGTTACTTGTCGCTGG |
| qPCR.AtATG10.Re | TTCTCCACCGCTGCAGTATC |
| qPCR.AtATG12a.Fw | TGTGAATAGTGCTTTCTCGCCAA |
| qPCR.AtATG12a.Re | CAACAAAGCAAGCATCTAGCG |
| qPCR.AtATG12b.Fw | TCCTCCAAAATCGTTCTTCTCAAG |
| qPCR.AtATG12b.Re | TCACATAAACAAACAATGAGTCAGA |
| qPCR.AtATG3.Fw | TCATCCACACTTGCCTGGTA |
| qPCR.AtATG3.Re | CCGAGATCAAAGTCCATTGTG |
| AtATG8a-qPCR-Fw | TCGATCTTTGGATGACTTTG |
| AtATG8a-qPCR-Re | GCAATGTACATCTTTATCTT |
| ATG8s-qPCR-Fw * | CCAACTGCTGCAATGATGTCTG |
| ATG8s-qPCR-Rv* | CCAAAGGTGTTCTCTCCACTGTA |

*primers anneal on a conserved region of *ATG8* genes, leading to amplification of multiple *ATG8* orthologs.
