# Supplementary material for: Transcriptional stimulation of rate-limiting components of the autophagic pathway improves plant fitness
Source: J Exp Bot. 2018 Jan 20;69(6):1415–32. doi: 10.1093/jxb/ery010 (PMC6019011; doi:10.1093/jxb/ery010)
Supplement: Supplementary Figures [file ery010_suppl_supplementary_figures.pdf]

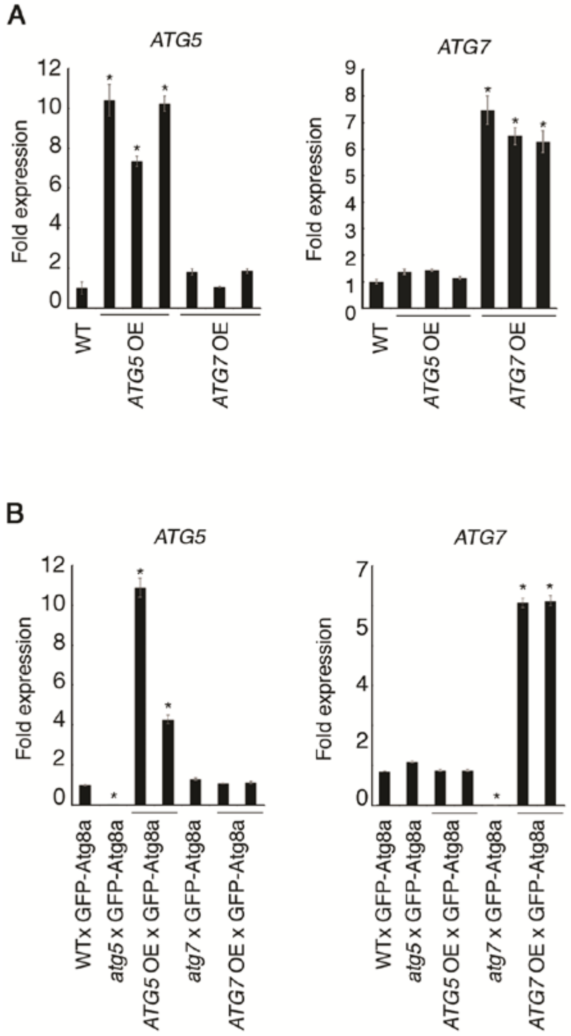

Figure S1. qRT-PCR analysis of *ATG5* and *ATG7* transcripts in WT and *ATG*-overexpressing plants.

(**A**) A representative qRT-PCR performed on cDNA obtained from 10-day-old seedlings of WT and three individual *ATG5*-overexpressing (*ATG5* OE) or *ATG7*-overexpressing (*ATG7* OE) lines grown under normal conditions (150 µE m^{-2} s^{-1} light, 16 h photoperiod). Data represent mean ± S.D. for individual plants normalized to two reference genes (*PP2A* and *HEL*) and to WT; n=3, technical replicates; *, P<0.0001; *vs* WT, Dunnett's test.

(**B**) qRT-PCR performed on cDNA obtained from material used in Figure 2E. Data represent mean ± S.D. for 15-20 pooled seedlings of each genotype normalized to two reference genes (*PP2A* and *HEL*) and to WT; n=3, technical replicates; *, P<0.0001; *vs* WTxGFP-Atg8a, Dunnett's test.

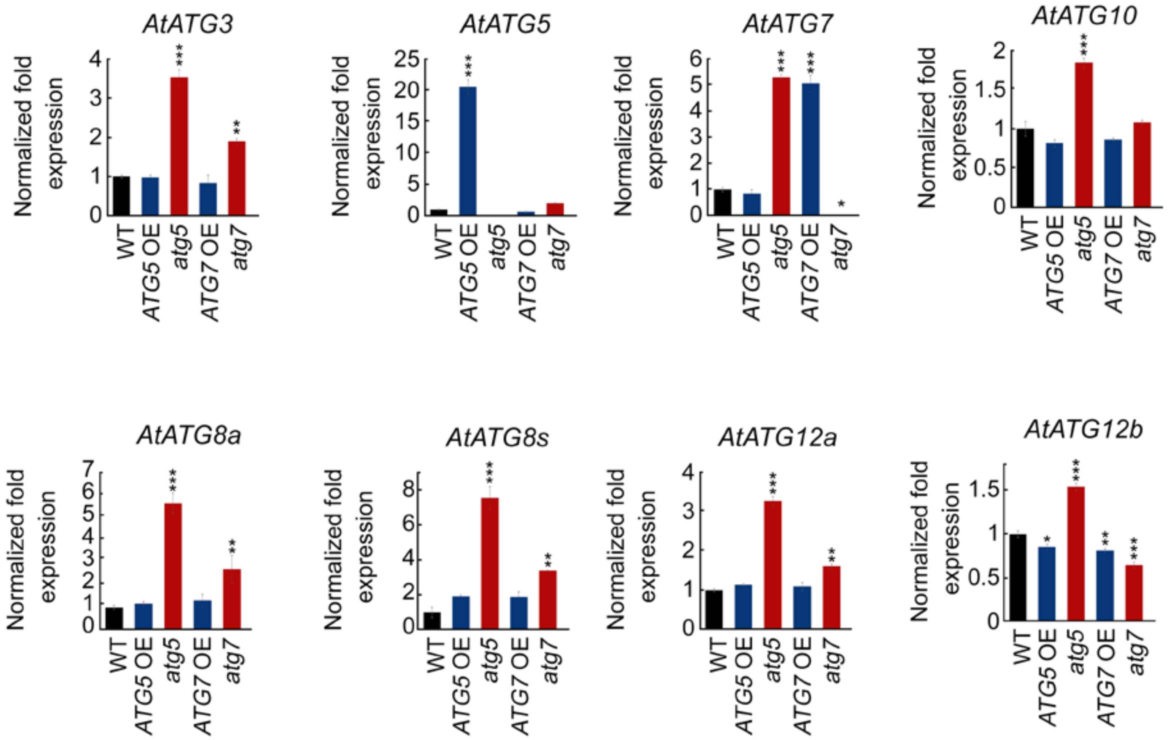

Figure S2. Overexpression of either *ATG5* or *ATG7* does not influence transcription of other components of the ubiquitin-like conjugation systems.

qRT-PCR was performed on the same leaf material as in **Figure 1B**. Data represent mean ± SEM for each genotype, normalized to two reference genes (*PP2A* and *HEL*) and to the WT; n=6. The experiment was performed twice. ***, p<0.0001; ** p<0.001; *p<0.05; *vs* WT, Dunnet's test. Red, *ATG*-knockouts; blue, *ATG* OE; black, WT.

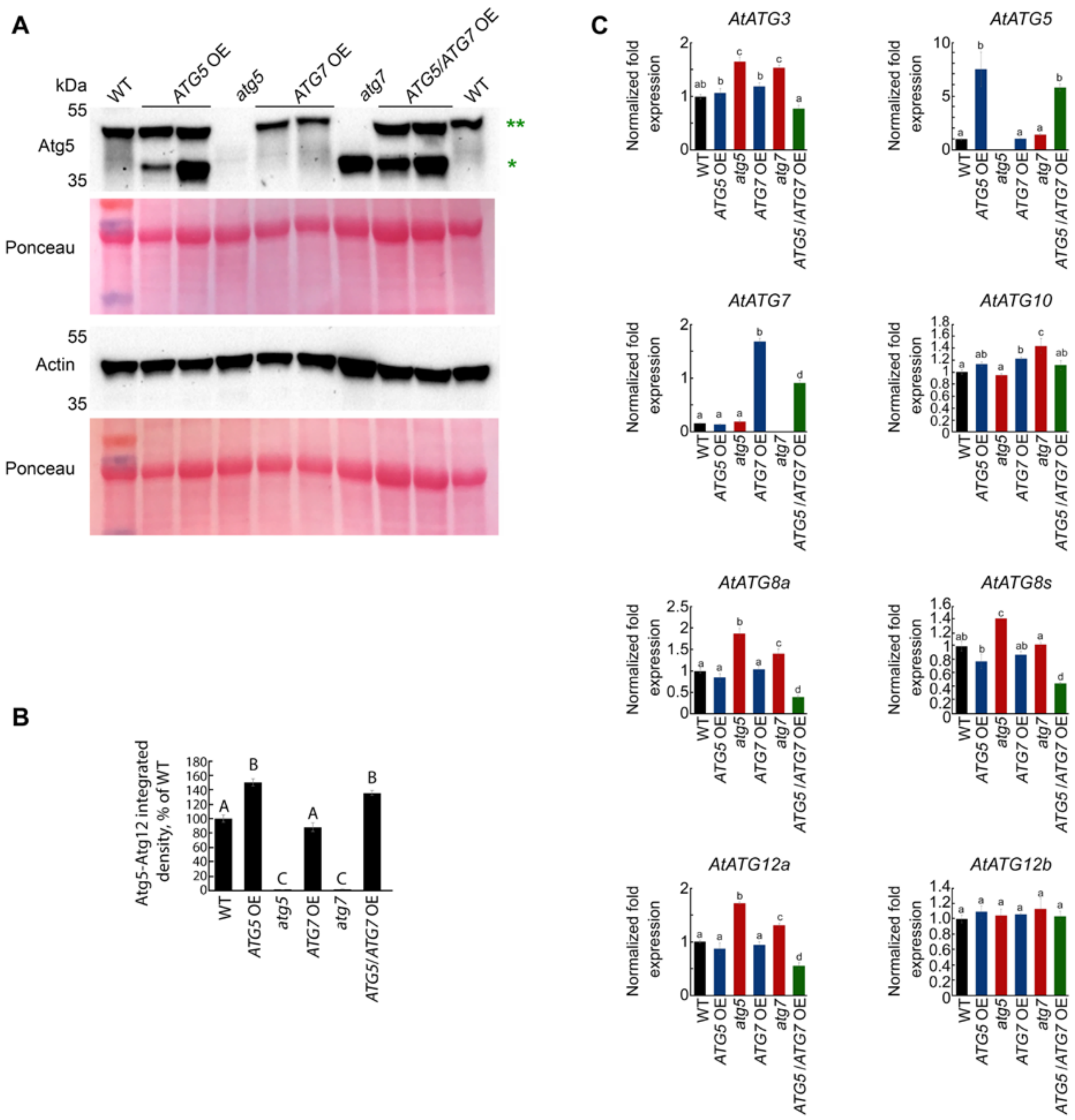

Figure S3. Simultaneous overexpression of *ATG5* and *ATG7* has the same effect on Atg5-Atg12 conjugation as overexpression of *ATG5* only and causes transcriptional suppression of other components of the two ubiquitin-like conjugation systems.

(**A**) Atg5, but not Atg7, is a limiting component in Atg12-Atg5 conjugation pathway. Western blot detection of Atg5 in rosette leaves of Col-0 WT, two individual *ATG5*-, *ATG7*-, *ATG5/ATG7* overexpressing lines (*ATG5* OE, *ATG7* OE and *ATG5/ATG7* OE, respectively), and *ATG5*- or *ATG7*-knockout (*atg5* and *atg7*, respectively) mutants. *, Atg5; **, Atg12-Atg5 conjugate. The

experiment was repeated twice, using two individual lines of each overexpressor background. Western blot detection of actin and Ponceau staining were used as loading controls.

(**B**) Densitometry of Atg5-Atg12 conjugate in (**A**). Integrated density for bands corresponding to the conjugate were first normalized to the values of corresponding actin bands and then expressed at % of WT. Data represent mean ± SEM; n=6. Mean values denoted by the same letter do not differ significantly at $P < 0.001$ (Student’s t-test).

(**C**) A representative qRT-PCR performed on cDNA obtained from rosette leaves of plants at the 0 DAF. Plants of indicated genotypes were grown under normal conditions (150 µE m^{-2} s^{-1} light, 16 h photoperiod). Data represent mean ± SEM for each genotype normalized to two reference genes (*PP2A* and *HEL*) and to WT; n=6. Mean values denoted by the same letter do not differ significantly at $P < 0.001$ (Student’s t-test). Red, *ATG*-knockouts; blue, *ATG* OE; black, WT.

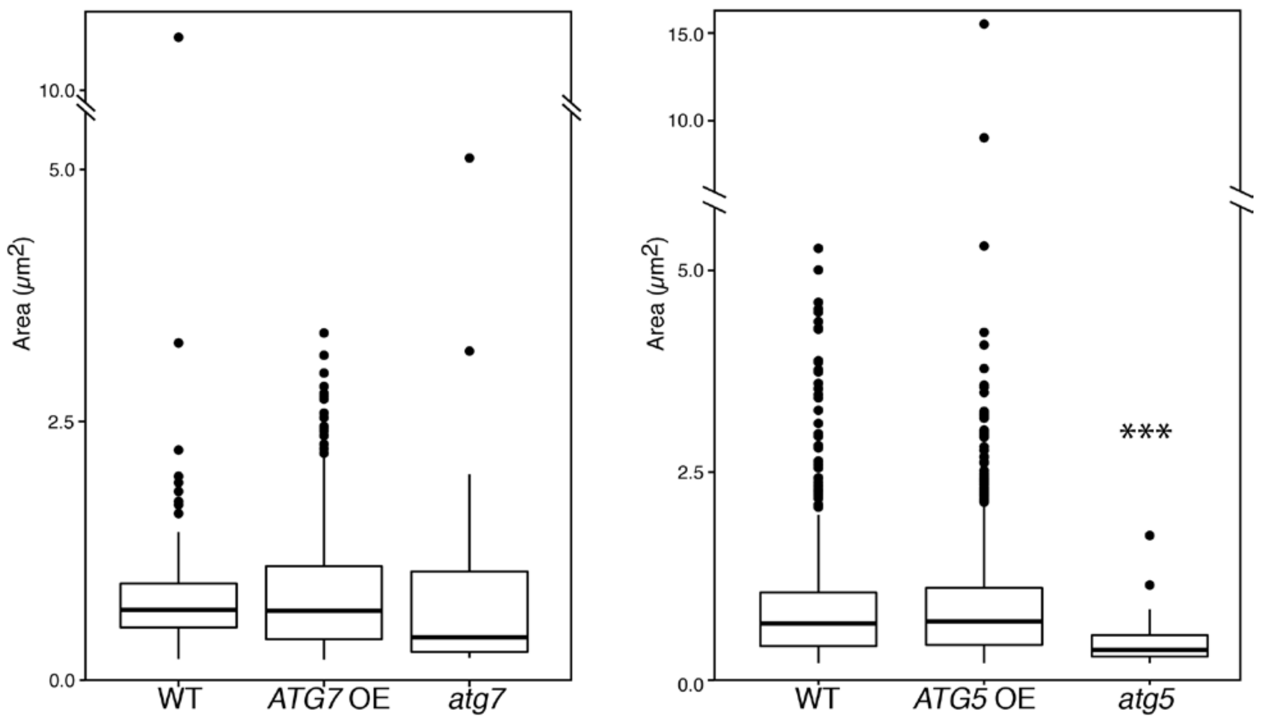

Figure S4. Size of GFP-positive puncta measured in WT, *ATG*-overexpressing and *ATG*-knockout backgrounds expressing GFP-Atg8a.

The box–plot diagram shows size of puncta measured on the same images as used for Figure 2B and exampled in Figure 2A.

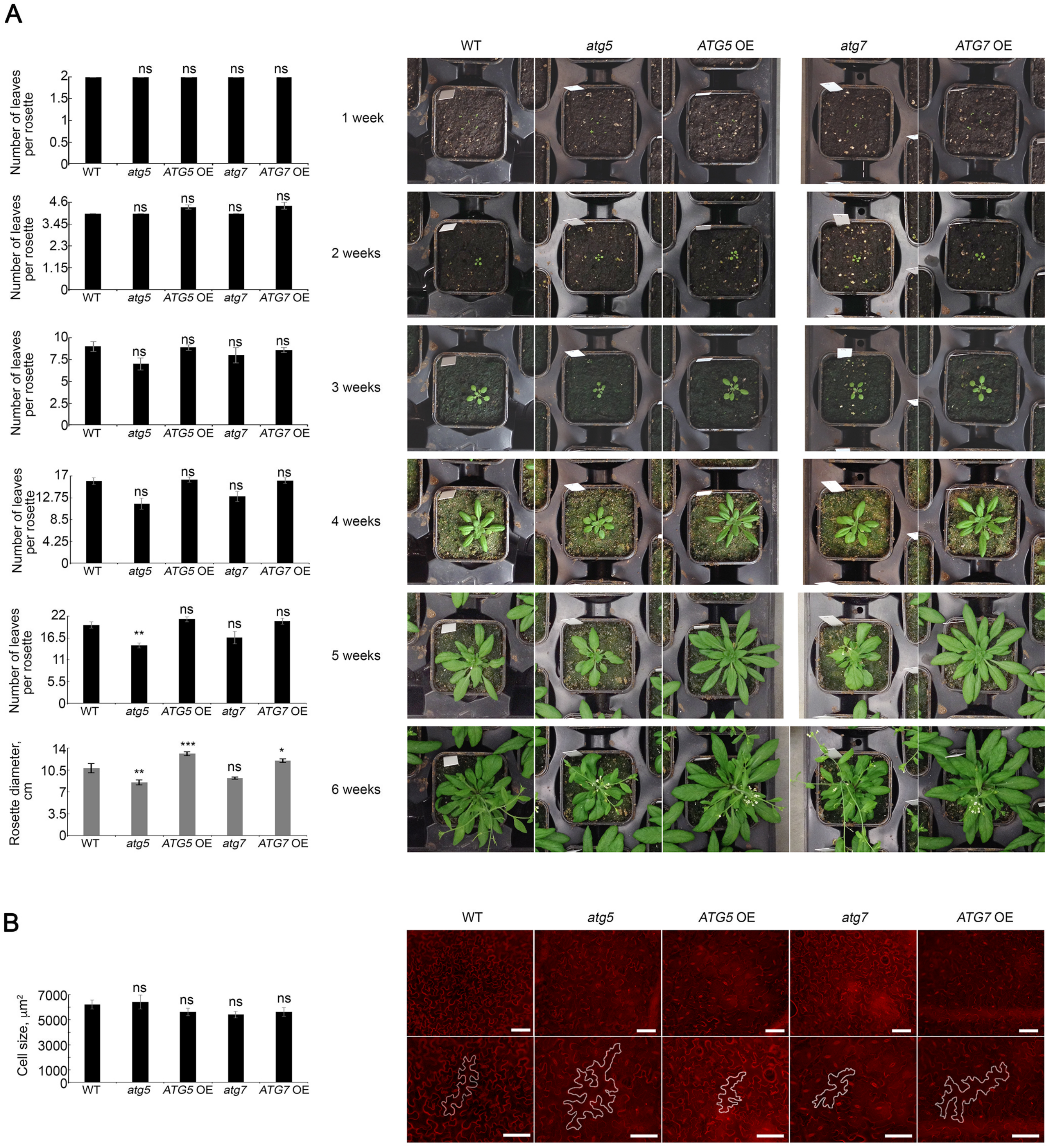
A
WT
atg5
ATG5 OE
atg7
ATG7 OE
1 week
2 weeks
3 weeks
4 weeks
5 weeks
6 weeks
Number of leaves per rosette
Rosette diameter, cm
ns
**
***
*
B
Cell size, μm²
7000
5000
3000
1000
0

Figure S5. Overexpression of *ATG5* or *ATG7* does not influence number of rosette leaves or cell size.

Three independent lines overexpressing *ATG5* or *ATG7* were used for the experiment. Plants were grown in individual pots placed into trays at random positions; all genotypes were represented in each tray. Trays were kept under normal conditions (150 µE m^{-2} s^{-1} light, 16 h photoperiod) and images were taken every 2-3 days.

(**A**) Phenotype of leaf rosette imaged every week for up to 6 weeks after sowing and quantification of rosette leaf number (during the first 5 weeks) and rosette diameter at 6 weeks after sowing. Data on charts represent mean ± S.D., n = 4-15. ns, non-significant; *, P<0.1; **, P<0.05; ***, P< 0.01; *vs* WT, Dunnett's test. Planting pots size was 8x8 cm.

(**B**) Representative images of epidermal rosette leaf cells stained with propidium iodide to visualize cell walls (top panel) and the same images magnified with an example of a cell selection used for quantification (bottom panel). Scale bars, 100 µm. Data on the chart represent mean ± S.D., n = 86-92 cells. ns, non-significant; Dunnett's test *vs* WT.

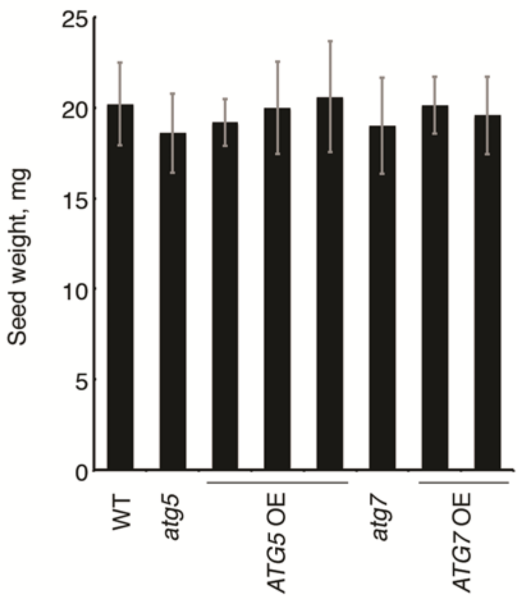

Figure S6. Overexpression of *ATG5* or *ATG7* does not influence the weight of an individual seed.

Seeds were harvested from plants of WT, *ATG*-knockouts (*atg5* and *atg7*), three individual *ATG5*-overexpressing (*ATG5* OE) lines and two individual *ATG7*-overexpressing (*ATG7* OE) lines grown under normal conditions. Data represent mean ± S.D. for at least 9 plants. Dunnett's test *vs* WT revealed no statistically significant differences between the mean values.

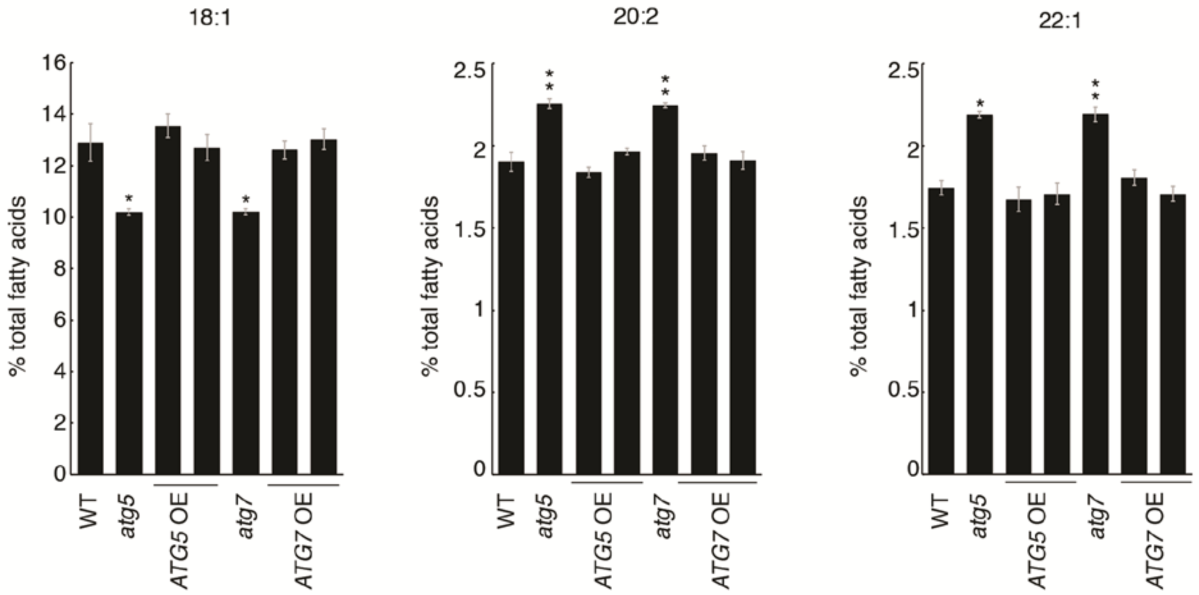

Figure S7. Knockout but not overexpression of *ATG5* or *ATG7* alters the composition of seed fatty acids. 18:1, oleic acid; 20:2, eicosa-13,16-dienoic acid; 22:1, erucic acid. Data represent mean ± SEM, n=3. **, P<0.0005; *, P<0.005; *vs* WT, Dunnett's test.
