# Supplementary material for: Transcriptional stimulation of rate-limiting components of the autophagic pathway improves plant fitness
Source: J Exp Bot. 2018 Jan 20;69(6):1415–32. doi: 10.1093/jxb/ery010 (PMC6019011; doi:10.1093/jxb/ery010)
Supplement: Supplementary Data [file ery010_suppl_supplementary_data.pdf]

OakLabs

Genome-wide Gene Expression Analysis Service

Contact details:

Neuendorfstraße 16b
D-16761 Hennigsdorf
Germany

Email: service@oak-labs.com
Phone: +49-3302 / 2 80 09 45

Version 7.0

This guide will give you an overview of all relevant procedures for genome wide gene expression analysis including the optional data analysis and will help you to make the most out of your data. Please contact our service team in case of any questions.

Contents

1 Summary of the Delivered Data **3**

2 Laboratory Workflow **3**

2.1 RNA Quality Control . . . 3

2.1.1 Labelling Reaction . . . 4

2.1.2 Hybridisation and Microarray Wash . . . 4

2.1.3 Scanning Microarray . . . 4

2.1.4 TIF Processing . . . 4

3 Data Analysis **4**

3.1 Data of Raw and Normalised Signals . . . 4

3.2 Quality Assurance . . . 7

3.2.1 Box Plots . . . 7

3.2.2 Hierarchical Clustering . . . 8

3.2.3 Correlation Analysis . . . 9

3.2.4 Principal Component Analysis . . . 10

3.3 Statistical Analysis . . . 11

3.3.1 Fold Change . . . 11

3.3.2 P-Value . . . 11

3.3.3 Controlling the False Discovery Rate . . . 12

3.3.4 Underlying Computations . . . 13

3.4 Data Visualisation . . . 15

3.4.1 Heat Maps . . . 15

3.4.2 Volcano Plots . . . 16

4 Closing Remarks **17**

1 Summary of the Delivered Data

- Laboratory workflow
  - Results of the Bioanalyzer (section 2.1)
  - Summary file listing quality control values for all samples (section 2.1.1)
  - Data extraction
- Data analysis
  - Data of raw and normalised signals (.txt files, section 3.1)
  - Figures of quality assurance (.pdf plots)
    * box plots (section 3.2.1)
    * hierarchical clustering (section 3.2.2)
    * principal component analysis (section 3.2.4)
    * pearson correlation analysis (section 3.2.3)
  - List of differentially expressed genes (.txt files, section 3.3)
  - Plots of data visualisation (.pdf plots)
    * Heat maps (section 3.4.1)
    * Volcano plots (section 3.4.2)
  - Pathway analysis and visualisation

2 Laboratory Workflow

2.1 RNA Quality Control

After receipt of your RNA samples, all of them undergo a quality control to determine the quality and quantity of the total RNA. The quality control is done via the 2100 Bioanalyzer (Agilent Technologies) using the RNA 6000 Pico Kit for total RNA samples. For quantity control, a photometrical measurement with the Nanodrop 2000 spectrophotometer (Thermo Scientific) is used. A sample's quality is evaluated based on the Bioanalyzer's RNA integrity number (RIN) and the two disctinct peaks representing the 18S and 28S rRNA as well as the overall electropherogram. After successfully passing quality control, RNA samples are subjected to further processing.

Bioanalyzer files are provided with your data delivery and visualise each sample in a gel image and an electropherogram.

2.1.1 Labelling Reaction

For genome-wide gene expression analysis on OakLabs' ArrayXS Agilent microarrays as well as on Agilent's catalogue microarrays, OakLabs fulfills the requirements for certified service providers and follows strictly the relevant protocols. For labelling, the Low Input QuickAmp Labeling Kit (Agilent Technologies) is used to generate fluorescent cRNA (complementary RNA). For 1st strand synthesis, either oligo-dT primer or a random primer / oligo-dT primer mixture (WT primer) is used. After 2nd strand synthesis, an in vitro transcription for synthesis of cRNA labelled with cyanine 3-CTP is performed. Please refer the summary file for details regarding the used primer, yield of cRNA as well as cy3 incorporation rate.

2.1.2 Hybridisation and Microarray Wash

For hybridisation, the Agilent Gene Expression Hybridisation Kit (Agilent Technologies) is used. For example, 600 ng of each cRNA is hybridised on 8x60K microarrays at $65\,^{\circ}\mathrm{C}$ for 17 h using Agilent's recommended hybridisation chamber and oven. Afterwards microarrays are washed once with the Agilent Gene Expression Wash Buffer 1 for one minute at ambient temperature followed by a second wash with preheated ($37\,^{\circ}\mathrm{C}$) Gene Expression Wash Buffer 2 for one minute.

2.1.3 Scanning Microarray

Fluorescence signals on microarrays are detected by the SureScan Microarray Scanner (Agilent Technologies) at a resolution of 3 micron for SurePrint G3 Gene Expression Microarrays and 5 micron for HD Microarray formats, generating a 20 bit TIFF file.

2.1.4 TIF Processing

Agilent's Feature Extraction software version 11 is used to read and process the TIFF files. The most important columns in the resulting raw data output of 1-colour hybridisations (.txt format) are described in table 1.

The summary file lists the QC values for each sample as well as the file names of the according raw data.

3 Data Analysis

3.1 Data of Raw and Normalised Signals

Prior to normalisation of the background substracted signals, the following processing steps are performed:

- removal of signals from control probes
- mean of signals from replicate probes

- mean of signals from all probes of a target

Data of all samples are quantile normalised using the ranked mean quantiles according to Bolstad et al. "A comparison of normalisation methods for high density oligonucleotide array data based on variance and bias", Bioinformatics (19), 2003, Oxford Univ Press. Briefly, the mean signal of each target is ranked relative to all other targets. The ranked signal value is replaced with the mean quantile value of the same rank. Thus, the highest value in all samples becomes the mean of the highest values, the second highest value becomes the mean of the second highest values, and so on.

Raw and normalised data are provided in different sheets of one Excel file as well as in a .csv file which can easily be imported to Excel for further analysis.

Workflow for the import of data into an existing Excel worksheet:

1. Click the cell where you want to put the data from the text file.
2. On the **Data** tab, in the **Get External Data** group, click **From Text**.
3. In the **Import Data** dialog box, do the following:
   - Ensure that the **Delimited** option is selected and click **Next**.
   - Select **Tabstop** in the delimiters section. The text qualifier box should show the asterisk symbol. Click **Next**.
   - Make sure the data format of every column is set to **Standard** and click **Advanced** to specify the type of decimal and thousands separators that are used in the text file.
   - Please select, a point for the **Decimal separator** and a comma for the **1,000 separator**. Click **Next** and **Finish**.

Table 1 – Description of the most important columns of the raw data output.

Column (g=green)	Description
ProbeName	An Agilent-assigned identifier for the probe synthesized on the microarray.
GeneName	This is an identifier for the gene for which the probe provides expression information. The target sequence identified by the systematic name is normally a representative or consensus sequence for the gene.
SystematicName	This is an identifier for the target sequence that the probe was designed to hybridize with. Where possible, a public database identifier is used (e.g., TAIR locus identifier for Arabidopsis). Systematic name is reported ONLY if Gene name and Systematic name are different.
gProcessedSignal	The signal left after all the FE processing steps have been completed. In the case of one colour, ProcesssedSignal contains the Multiplicatively Detrended BackgroundSubtracted Signal if the detrending is selected and helps. If the detrending does not help, this column will contain the BackgroundSubtractedSignal.
gMedianSignal	Raw median signal of feature in green channel (inlier pixels)
gBGMeanSignal	Mean local background signal (local to corresponding feature) computed per channel (inlier pixels)
gBGSubSignal	= Background-subtracted signal
gIsPosAndSignif	Boolean flag, established via a 2-sided t-test, indicates if the mean signal of a feature is greater than the corresponding background and if this difference is significant. 1 indicates feature is positive and significant above background.
gIsWellAboveBG	Boolean flag indicating if a feature is WellAbove Background or not, feature passes gIsPosAndSignif and additionally the gBGSubSignal is greater than 2.6*gBGSDUsed.

3.2 Quality Assurance

Quality control of data is an essential step when performing high quality gene expression analysis. The concept used for ascertaining data quality in genome-wide gene expression studies helps you to identify outlier samples and to decide whether a sample needs to be removed from the data set.

The results of several tools are considered to identify potential outlier samples. A repeated analysis is performed without those samples. The delivered data contain both analysis results, with and without potential outlier samples.

3.2.1 Box Plots

Box plots are useful to quickly visualise the variation within one data set and between data sets. The key features of a box plot are the median (black line) and the variability (inter-quartile range) indicated by the 75th percentile (top edge of box) and 25th percentile (bottom edge of box). Boxplots of data distributions before and after normalisation allow to evaluate if normalisation was effective as well as to identify potentially problematic samples (figure 1).

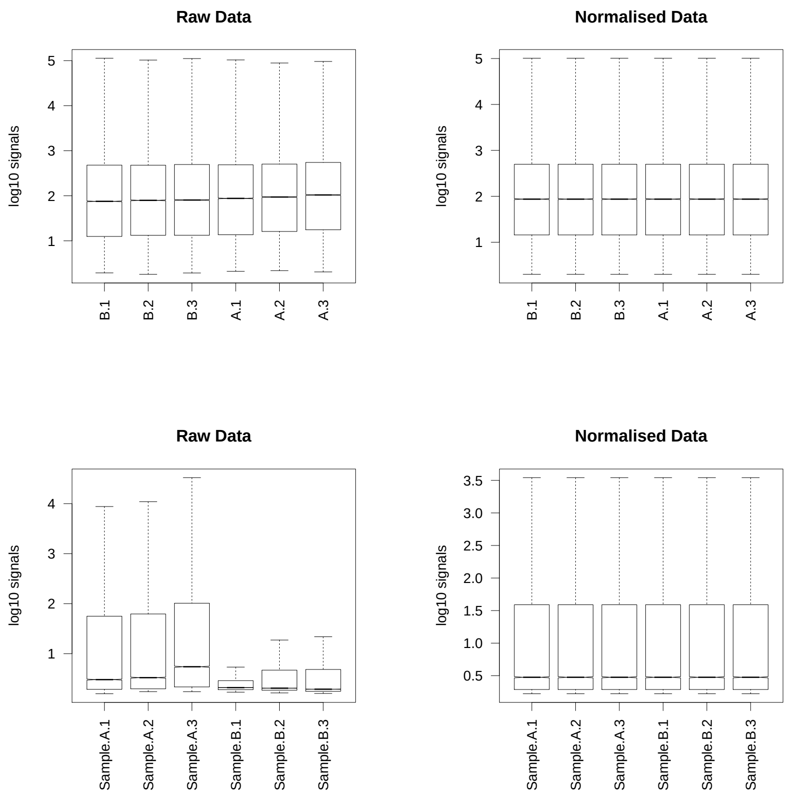

Figure 1 – Boxplots. The boxplots are from data before (left) and after normalisation (right). The data in the upper boxblots have a very similar distribution, even before normalisation. In contrast, the lower boxplots illustrate that the signal distribution for samples of group B is very narrow and the median is relatively low. Though the normalisation is effective, special attention should be paid to the suspicious samples.

3.2.2 Hierarchical Clustering

The overall performance of an experiment can be evaluated by clustering samples using the correlation metric. The result of hierarchical clustering is a graph called a dendrogram (see figure 2). The clustering is based on normalised expression values.

Samples that have the most similar expression profiles are clustered together. Therefore, hierarchical clustering is useful for identifying outlying samples.

Technical replicates (e.g. hybridisation) are expected to be the most similar followed by biological replicates from the same origin (e.g. heart tissue from different individuals). Samples may not cluster necessarily according to their biological category, e.g. when samples in an experiment show few differences in gene expression levels. In those cases, samples lack sufficient differences to segregate into different clusters.

Figure 2 depicts examplary plots of hierarchical clusterings with one plot illustrating a potential outlier sample. If potential outlier samples are observed the data analysis is repeated excluding the affected sample(s).

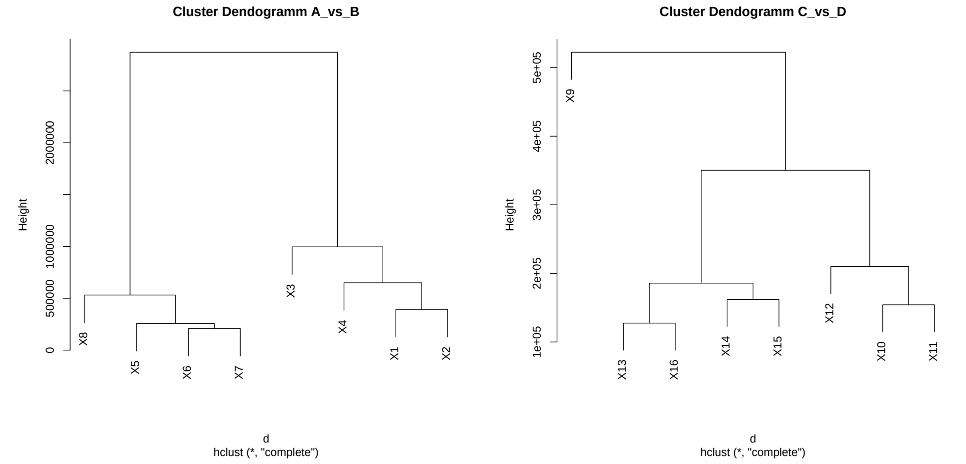

Figure 2 – Hierarchical Clustering. The dendrograms show the results of a hierarchical clustering. Samples with similar expression patterns are located close to each other. The left plot illustrates that samples of treatment A (X1-X4) form a cluster and samples of treatment B (X5-X8) form a second cluster. In the right plot, sample X9 is dissimilar from the other samples and does not enter a cluster. Sample X9 is a potential outlier.

3.2.3 Correlation Analysis

Replicate samples usually show high correlation. As described in section 3.2.2, correlation between samples of the same group is expected to be at least as high as the correlation of samples belonging to different groups. A correlation analysis is valuable to form an opinion of the quality of the data (see figure 3).

The Pearson correlation coefficient is a measure of the linear correlation (dependence) between the data of two samples, giving a value between +1 and -1 inclusive, where 1 is total positive correlation, 0 is no correlation, and -1 is total negative correlation.

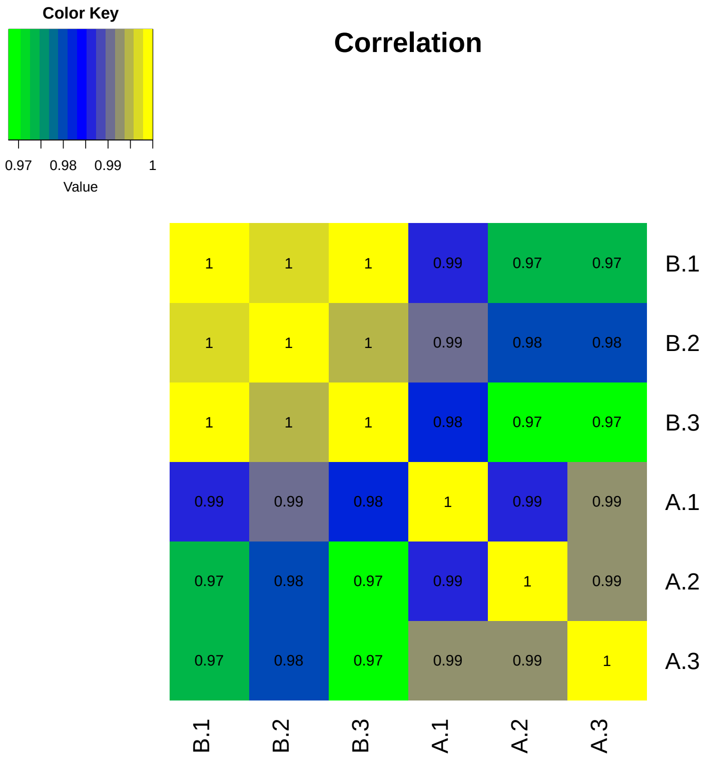

Figure 3 – Pearson Correlation heat map with correlation coefficients.

3.2.4 Principal Component Analysis

Objectives of principal component analysis (PCA) are to discover or to reduce the dimensionality of the data set and to identify the highest variabilities (principal components) of the data. The data is visualised in a two dimensional coordinate system, where both axes represent the two highest variabilities of the data. The labels on the axes show the relative wheights (in percentage) for the first component (x-axis) and the second component (y-axis).

Similiar to a hierarchical clustering algorithm, we can observe in this plot whether or not the distance of samples within one group is bigger than the distance between samples of different groups.

Given the signal intensity data of a microarray experiment involving N genes and K samples, this data can be conceptualised as K data points in an N dimensional space. Imagination or visualisation is hardly possible, even for a small microarray experiment with four samples. When performing PCA, a new system of coordinates is computed. The new system is composed out of the principal components of the covariance matrix. The axes are ordered according to their eigen values such that the first axis represents the combination with the highest variance in the data. Usually the first two principal components give a good impression of how the data clusters according to its variance.

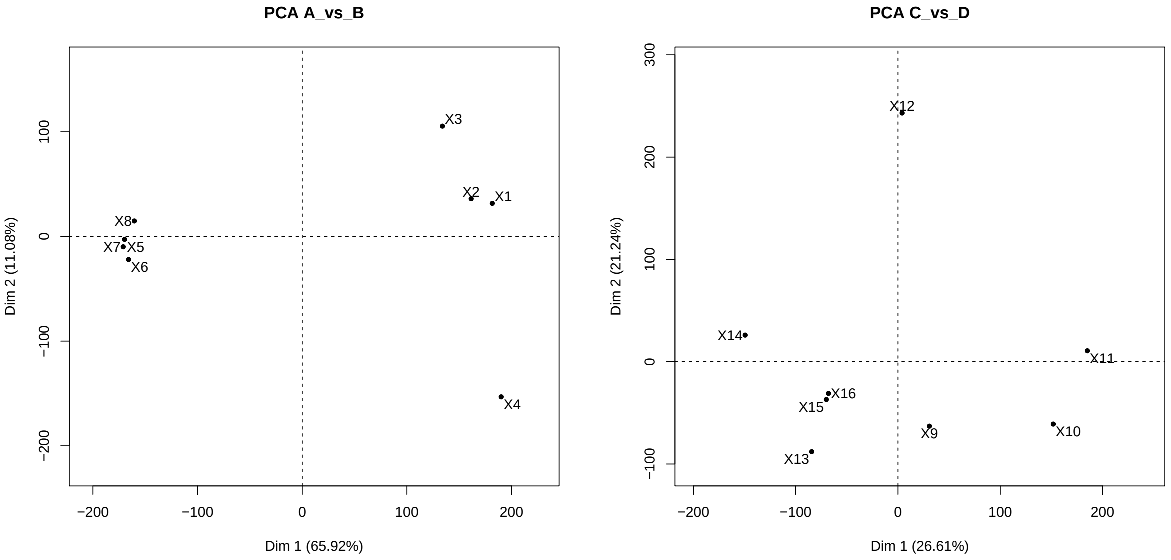

Figure 4 – Principial Component Analysis. The plots show the results of a principal component analysis. In the left plot the PCA successfully separates two clusters for both treatments (treatment A: X1-X4; treatment B: X5-X8). In contrast, the PCA depicted in the right plot could not separate the treatments (treatment C: X13-X6; treatment D: X9-X12) very well as the cumulative variance of principal component 1 and 2 is less than 50%.

3.3 Statistical Analysis

Samples that are compared are normalised together prior to the statistical analysis (see section 3.1 for details). Statistical parameters are computed if the samples are organised in at least two groups where each group contains biological replicates. The high reproducibility of data obtained with Agilent technology allows to avoid technical replicates completely.

3.3.1 Fold Change

Based on the normalised data, expression ratios are calculated (see section 3.3.4 for details) to represent expression differences in a very intuitive manner. For example, genes that do not differ in their expression level will have an expression ratio of approximately 1. However, this representation may not be helpful if one has to represent up-regulation and down-regulation. For example, a gene that is up-regulated in a mutant compared to the wildtype by a factor of 4 has an expression ratio of 4

$$\begin{aligned} \text{Gene}_{\text{mut}} &= 4 \times \text{Gene}_{\text{wt}} \\ \Rightarrow \frac{\text{Gene}_{\text{mut}}}{\text{Gene}_{\text{wt}}} &= 4 \end{aligned}$$

However, if a gene is down-regulated by a factor of 4, the expression ratio becomes 0.25.

$$\begin{aligned} 4 \times \text{Gene}_{\text{mut}} &= \text{Gene}_{\text{wt}} \\ \Rightarrow \frac{\text{Gene}_{\text{mut}}}{\text{Gene}_{\text{wt}}} &= 0.25 \end{aligned}$$

Thus, up-regulation is mapped between 1 and infinity, whereas down-regulation is mapped between 0 and 1.

To eliminate this inconsistency in the mapping interval, a logarithmic base 2 transformation is performed (i.e. $\log_2$ (expression ratio)). This has the major advantage that it treats differential up-regulation and down-regulation equally with a continuous mapping space. For example, if the expression ratio is 1, then $\log_2(1.0) = 0$ represents no change in expression. If the expression ratio is 4, then $\log_2(4.0) = 2.0$ and for expression ratio of $\log_2(0.25) = -2.0$. Thus, the mapping space is symmetric and up-regulation and down-regulation are comparable.

Despite the advantages of the $\log_2$ fold changes, using expression ratios as a metric for gene expression means to remove information about gene signals of the genes. For example, genes that have mut/wt ratios of $400/100$ and $4/1$ have the same expression ratio of 4.

We recommend to keep the gene signals in view and not give much attention to genes with signals below 10 in both groups.

3.3.2 P-Value

A hypothesis test is performed for each gene which indicates whether the observed fold changes are indeed statistically significant. By default, a Welch's t-test (or unequal variances t-test) is used to test the hypothesis that the signals of a gene in two biological groups of samples (e.g. mutant and wildtype) have equal means. The Welch's t-test is more reliable when the two biological groups have unequal variances and unequal sample sizes.

In contrast, if the samples are paired units, e.g. prior and after medical treatment of each patient, paired t-tests have greater power than unpaired tests as biological variation between individual samples within one group is disregarded. Therefore, paired t-tests are used whenever the experimental setup allows.

A histogram of the p-values is generated to evaluate how the statistical t-test behaved across all hypotheses. Figure 5 shows two histograms. A typical histogram (left histogram in figure 5 shows a peak close to 0 indicating significantly differentially expressed genes with some potential false positives and a flat distribution along the bottom. A different shaped histogram (e.g. such as shown in the right histogram in figure 5) indicate a potential problem with the applied hypotheses test.

You can get an estimate of the number of significantly differentially expressed genes by looking at the peak on the left: the taller the peak, the more genes are significantly expressed.

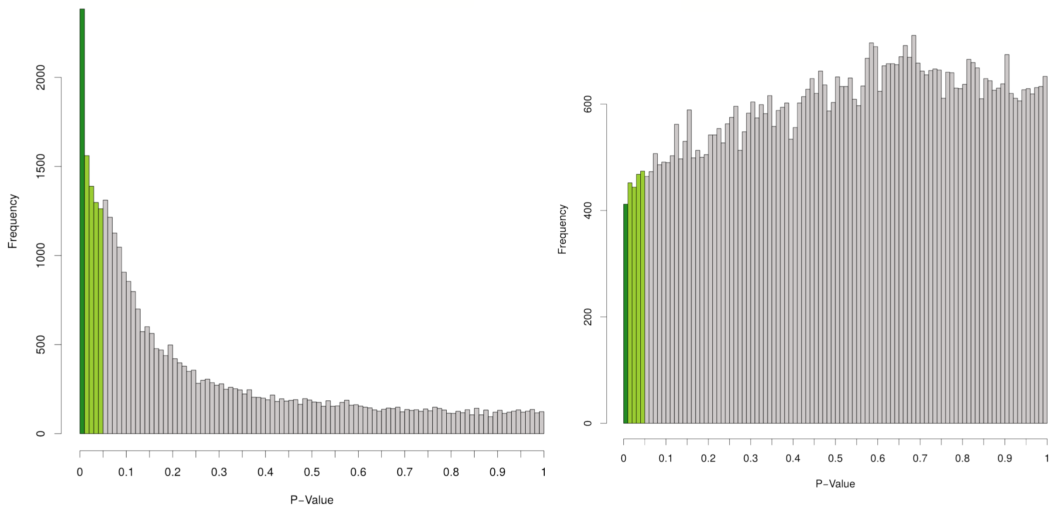

Figure 5 – Histogram of p-values. The plots show the histogram of p-values obtained for hypothesis tests of two biological groups of samples (e.g. mutant and wildtype). The left plot shows a typical histogram with a peak close to 0 (green) indicating significantly differentially expressed genes with some potential false positives and a flat distribution along the bottom, which are uniformly distributed between 0 and 1.

The significantly differentially expressed genes (based on the p-value and log_2 cutoff) stating the adjusted p-values at the level $q = 0.05$ are provided in a sheet in the Excel file as well as in a .csv file. The Excel file also contains the sheet “NoFilter” which gives p-values and log_2 for all genes.

3.3.3 Controlling the False Discovery Rate

The false discovery rate (FDR) is defined as the expected proportion of “false positives”, genes that are found to be statistically different between two groups.

The conventional setup where a significance level of α is chosen to control a “false positive” for each hypothesis, is not suitable to control the FDR for the whole experiment. Multiple hypothesis

testing ensures that the overall false positive rate among the significantly expressed genes is less than a user specified global significance level of q.

For a list of 50,000 genes which have been tested at a significance level of $\alpha = 0.05$, up to 2499 genes might be (but not have to be) "false positives" - independently on the number of identified significantly differentially expressed genes.

In contrast, multiple hypothesis testing with an FDR at a level of $q = 0.05$ means that max. 5% of the genes that have been identified to be significantly differentially expressed are "false positives". While the absolute number of "false positives" depends on the number of significantly differentially expressed genes, the overall proportion of "false positives" is independent of it.

There are several strategies for multiple testing corrections which are more or less stringent. The more stringent a multiple testing correction, the less "false positive" genes are allowed. However, a stringent multiple testing correction means that the rate of "false negatives" (genes that are called non-significant when they are) is very high.

OakLabs adjusts the p-values according to the adaptive Benjamini-Hochberg procedure (1) which has been proposed by Benjamini and Hochberg (2) to increase the statistical power, that is to decrease the number of "false negatives".

In the Benjamini-Hochberg procedure, the p-values are first sorted and ranked. The smallest p-value gets rank 1, the second rank 2, and so on. Then, each p-value is multiplied by the number of genes analysed on the microarray and divided by its assigned rank to give the adjusted p-values. If the adjusted p-value is below the threshold (default $q < 0.05$) the gene is considered to be significant.

In the adaptive Benjamini-Hochberg procedure, instead of multiplying the p-value by the number of genes on the microarray, the estimated number of genes that are not significantly differentially expressed is used. The estimate is derived using a linear fit of the underlying data.

As the adjusted p-values in the adaptive procedure are smaller compared to the Benjamini Hochberg procedure, the adaptive FDR procedure increases the statistical power.

The default p-value threshold is 0.05 and $\log_2$ fold change < -1 or > 1. All genes meeting those criteria are provided in a table as significantly differentially expressed genes. The genes are listed in descending order according to the fold change. Please keep in mind: $\log_2$ equals 1 for 2 fold up-regulated genes and $\log_2$ equals -1 for 2 fold down-regulated genes.

3.3.4 Underlying Computations

Suppose the microarray experiment consists of two groups A, B. Group A has N_A samples and group B consists of N_B samples. We then compute the $\log_2$ values for all elements in each group

$$L_A = \left\{ \log_2(a_i) \;\middle|\; \forall a_i \in A \right\} \tag{1}$$

$$L_B = \left\{ \log_2(b_i) \;\middle|\; \forall b_i \in B \right\} \tag{2}$$

The mean and standard deviations of the two sets L_A, L_B can now be compared with each other using a significance test. It is important to perform the log transformation prior to the significance test, as this usually implies that the data is taken from a population which follows a normal distribution. We are using a two-sample t-test with unequal variances, also known as *Welch's*

t-test. The t-statistic is then given by

$$t = \frac{\overline{L_A} - \overline{L_B}}{\sqrt{\frac{s^2_{L_A}}{N_A} + \frac{s^2_{L_B}}{N_B}}} \tag{3}$$

$$df = \frac{\left(\frac{s^2_{L_A}}{N_A} + \frac{s^2_{L_B}}{N_B}\right)^2}{\frac{s^2_{L_A}}{N_A^2(N_A-1)} + \frac{s^2_{L_B}}{N_B^2(N_B-1)}} \tag{4}$$

$s^2_{L_A/L_B}$ is the variance of the two groups. The underlying distribution can be approximated by the usual t-distribution by applying equation 4 for the degree of freedom.

In order to compute the significance, we compute the p value which gives the probability that the measured value lies even farther in the tail of the distribution. If the p value is smaller than 0.05 (5%) we indicate that the difference in the mean $\log_2$ values is statistically significant.

Furthermore, we ignore all $\log_2$ values which lie between -1 and 1. That means our output file contains all targets which fulfil the following conditions:

$$p - \text{value} < 0.05 \tag{5}$$

$$\left(\overline{L_A} - \overline{L_B}\right) > +1 \text{ or} \tag{6}$$

$$\left(\overline{L_A} - \overline{L_B}\right) < -1 \tag{7}$$

3.4 Data Visualisation

3.4.1 Heat Maps

For the top 50 and top 100 significantly differentially expressed genes (based on p-values), heat maps have been created. A heat map is a false color image with a dendrogram added to the left side and to the top. A reordering of the rows (genes) and columns (samples) according to some set of values (row or column means) within the restrictions imposed by the dendrogram is carried out. The distance matrices of both dendrograms are calculated with the euclidean norm. For the colour representation, z-scores are calculated for each line. The z-score is the difference of a gene's normalised signal of one sample and the gene's mean signal of all samples divided by the standard deviation. The absolute value of z represents the distance between a sample's gene signal and the gene's mean signal of all samples in units of the standard deviation. z is negative (blue) if the sample's gene signal is below the mean, positive (red) if above. Fig. 6 shows an exemplary heat map.

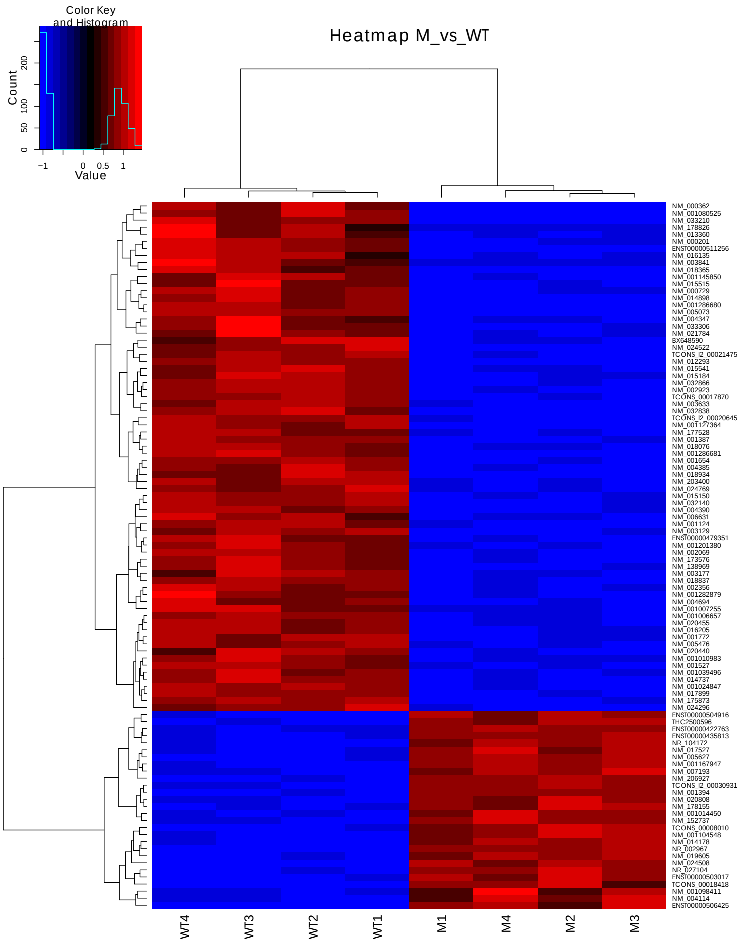

Figure 6 – Heat Map. False colour image of the top 50 significantly differentially expressed genes.

3.4.2 Volcano Plots

A volcano plot arranges genes along dimensions of biological and statistical significance. The horizontal axis is the $\log_2$ fold change between the two groups of samples, e.g. mutant versus wildtype, and the vertical axis represents the p-value (on a negative log 10 scale: the smaller the p-values the larger the -log10 p-value). The horizontal axis indicates biological impact of the change; the vertical axis indicates the statistical evidence of the change. Names are attached to the top genes in terms of fold change and p-value of indicated potentially most promising candidates for follow-up studies. Fig. 7 shows an exemplary volcano plot.

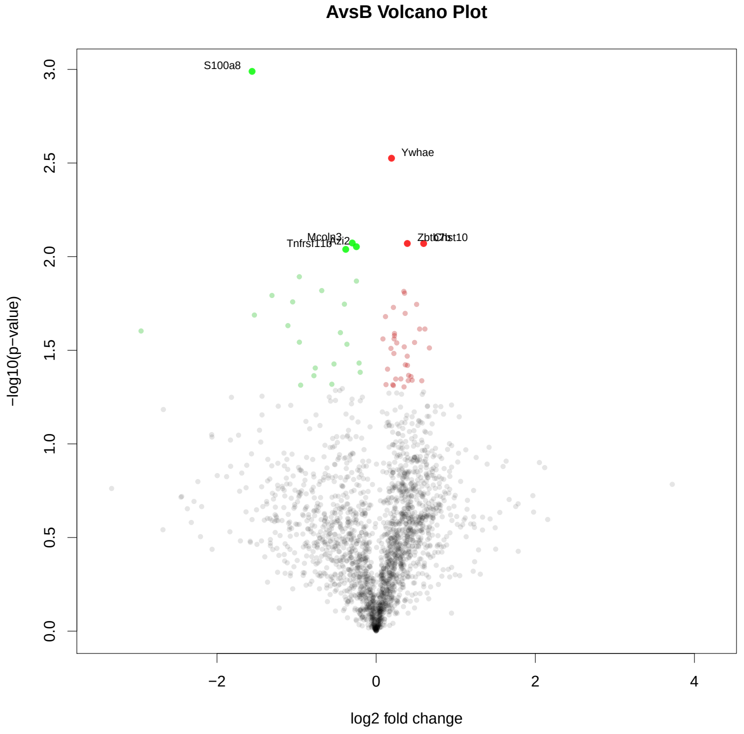

Figure 7 – Volcano plot. Significantly up-regulated genes are visualised as red dots, down-regulated as green dots. Genes with p-values < 0.01 appear intensely green and red, respectively.

4 Closing Remarks

No matter whether you have decided for Agilent's catalogue microarrays or OakLabs' ArrayXS Agilent microarrays for gene expression profiling, you can enjoy the following advantages:

- with the large dynamic range over five orders of magnitude you can access the full spectrum of transcripts
- the high reproducibility of the technology allows you to avoid technical replicates and to compare data obtained at different time points
- OakLabs' data analysis and pathway visualisation service facilitates the interpretation of your data

OakLabs' ArrayXS Agilent microarrays provide even additional value:

- experimental validation of each probe on ArrayXS guarantees a superior data quality
- parallel analysis of mRNA and non-coding transcripts from total RNA ensures that you receive comprehensive expression data without limitations

Like every statistical analysis, the results from your project will gain statistical reliability and significance with increasing numbers of samples. OakLabs Array-to-Go service is a cost efficient strategy to improve the statistical power of your data and to focus the analysis on up to 2,000 relevant genes.

Array-to-Go was specifically developed for the needs of researchers who already know the genes which are important for their study. Thus, it is possible to gain deeper insights into relevant pathways.

With Array-to-Go you can either choose from currently more than 90 preconfigured compilations or simply have your Array-to-Go custom-tailored for any species, e.g with the relevant genes which have been identified in the genome-wide gene expression profiling.

In comparison to qPCR panels, Array-to-Go offers a higher information value, while significantly less total RNA is required to detect lowly expressed genes.

We recommend to confirm expression data for candidate genes by quantitative realtime PCR as this is the gold standard in validating gene expression data. You can either use TaqMan qRT-PCR assays or choose SYBRgreen.

Please do not hesitate to contact us about our Array-to-Go or RT-qPCR service.

References

[1] Benjamini, Yoav and Hochberg, Yosef Controlling the false discovery rate: a practical and powerful approach to multiple testing Journal of the Royal Statistical Society. Series B (Methodological) JSTOR 1995.

[2] Benjamini, Yoav and Hochberg, Yosef On the adaptive control of the false discovery rate in multiple testing with independent statistics Journal of educational and Behavioral Statistics 2000.

[3] Gilbert, Peter B A modified false discovery rate multiple-comparisons procedure for discrete data, applied to human immunodeficiency virus genetics Journal of the Royal Statistical Society: Series C (Applied Statistics) 2005.
