# Supplementary material for: Transcriptional stimulation of rate-limiting components of the autophagic pathway improves plant fitness
Source: J Exp Bot. 2018 Jan 20;69(6):1415–32. doi: 10.1093/jxb/ery010 (PMC6019011; doi:10.1093/jxb/ery010)
Supplement: Supplementary Methods [file ery010_suppl_supplementary_methods.pdf]

Supplementary methods

Quantitative RT-PCR

One hundred milligrams of the sampled leaf material was used for RNA extraction. One microgram of RNA was used per RT reaction with Maxima kit (K1671, Fermentas, Thermo Fisher Scientific Inc). Several reference genes were selected from the list of genes stably expressed at different developmental stages of *Arabidopsis thaliana* (Czechowski *et al.*, 2005). Two of these genes *PP2A* (AT1G13320.1) and *RNA Helicase* (AT1G58050.1) were confirmed to be both stable at the selected developmental stages and therefore used for normalization. *ATG3*, *ATG5*, *ATG7, ATG8a, ATG8s, ATG10, ATG12a, ATG12b* and *NBR1* transcripts were detected using corresponding qPCR primers (**Table S5**). *ATG16* was not included into the list of analyzed components of the ubiquitin-like conjugation systems, due to the fact that its Arabidopsis orthologs have not yet been identified. qPCR reactions were performed in technical triplicates using IQ5 PCR Thermal Cycler (Bio-Rad) and DyNAmo Flash SYBR Green qPCR Kit (F415, Finnzymes, Thermo Fisher Scientific Inc). qRT-PCR data analysis was performed according to the comparative C_T method (Livak and Schmittgen, 2001) with qRT-PCR efficiency correction determined by the slope of standard curves. Fold-differences in transcript levels and mean standard error were calculated as described (Schmittgen and Livak, 2008).

Analysis of lipid content

Total lipid contents were measured as total fatty acids by converting the acyl groups into methyl esters and quantifying them by GLC. Seed samples (ca 2 mg) were homogenized in methanol/chloroform/0**.**15 M acetic acid containing 10 mM EDTA (2.5/1.25/0.9 mL)(Bligh and Dyer, 1959) using an Ultra Turrax® (IKA). After addition of 1.25 mL of chloroform and 1 mL of water and mixing, the extract was centrifuged and the lipid containing chloroform phase was redrawn. The chloroform phase was evaporated to dryness under nitrogen and the residue redissolved in 2 mL methylation solution (2% H_2SO_4 in water-free methanol) and methylated at 90 °C for 1 h. After methylation, 2 mL water and 2 mL hexane were added followed by brief vortexing and centrifugation. GC analysis of fatty acid methyl esters in the hexane phase was performed on a CP-wax 58 (FFAP-CB) column using a Shimadzu gas chromatograph. The identification of fatty acid methyl esters was performed by comparing the retention times with

authentic standards (Larodan). Quantification of fatty acid methyl esters was done by addition of heptadecanoic acid methyl esters as internal standard prior to methylation.

References

Bligh EG, Dyer WJ. 1959. A Rapid Method of Total Lipid Extraction and Purification. *Canadian Journal of Biochemistry and Physiology* **37**, 911-917.

Czechowski T, Stitt M, Altmann T, Udvardi MK, Scheible WR. 2005. Genome-wide identification and testing of superior reference genes for transcript normalization in Arabidopsis. *Plant Physiology* **139**, 5-17.

Livak KJ, Schmittgen TD. 2001. Analysis of relative gene expression data using real-time quantitative PCR and the 2(T)(-Delta Delta C) method. *Methods* **25**, 402-408.

Schmittgen TD, Livak KJ. 2008. Analyzing real-time PCR data by the comparative C-T method. *Nature Protocols* **3**, 1101-1108.
